# The AuTOMATIC trial: a multicentre digitally-automated, Bayesian, adaptive, parallel, factorial randomised controlled trial of SMS reminders for childhood vaccination

**DOI:** 10.1016/j.lanwpc.2026.101804

**Published:** 2026-02-11

**Authors:** Grace Currie, James Totterdell, Claire S. Waddington, Ian Peters, Alan Leeb, Gary Browne, Grahame Bowland, Katie Attwell, Catherine Hughes, Christopher C Blyth, Julie Marsh, Mark Jones, Tom Snelling

**Affiliations:** a School of Public Health, University of Sydney, Sydney, NSW, Australia; b Department of Clinical Sciences, Liverpool School Tropical Medicine, Liverpool, UK; c SmartVax, Illawarra Medical Centre, Perth, Australia; d Wesfarmers Centre of Vaccines and Infectious Diseases, The Kids Research Institute Australia, Perth, Australia; e Department of Social Sciences, University of Western Australia, Perth, Australia

**Keywords:** SMS reminders, Childhood vaccination, Nudge interventions, Trial automation, Learning health systems

## Abstract

**Background:**

The estimated effectiveness of SMS (short message service) reminders for improving childhood vaccine coverage and timeliness has varied in previous studies. The observed heterogeneity in effectiveness may be explained in part by variation in reminder content or timing of the reminder relative to the vaccine schedule date. We sought to evaluate the effectiveness of a range of SMS reminders of varied content and timing for improving on-time childhood vaccination.

**Methods:**

AuTOMATIC was a multi-centre Bayesian adaptive factorial randomised trial comparing four alternative SMS message framings at three alternative message timings versus a no reminder control strategy. Participants were parents of children registered with one of 20 primary care clinics Australia-wide and randomly assigned to one of 12 SMS reminder arms or to control. Reminders varied by framing of content (neutral, positive, risk-based or social benefit) and timing (14 days prior to the due date, on the due date, or 7 days afterwards). The primary endpoint was on-time vaccination, i.e. within 28 days of its scheduled date. Allocation probabilities were updated and stopping rules implemented over the trial according to pre-specified rules based on the posterior probability of effectiveness of each arm evaluated at interim analyses. Trial procedures were largely digitally automated. This trial was registered on Australian New Zealand Clinical Trials Registry (ACTRN12618000789268).

**Findings:**

Between January 14, 2021 and February 26, 2024, 9993 parents were randomised and all were included in the primary analysis; between 380 and 1110 were assigned to each of the 12 SMS reminder arms and 637 to control. The adjusted odds ratio (aOR) of on-time vaccination for each of the 12 SMS arms compared to control ranged from 1.02 [95% CrI 0.76–1.34] to 1.53 [1.22, 1.92] with a pooled effect aOR of 1.29 [1.06, 1.55]. This pooled effect corresponded to a standardised difference of 6% [2%, 11%] in the proportion of on-time vaccinations.

**Interpretation:**

On average, SMS reminders were associated with a modest increase in on-time vaccination compared to no reminder. There was evidence that neutral SMS reminders were less effective than persuasive reminders, but we were unable to identify a single best combination of reminder content framing and timing.

**Funding:**

10.13039/100009860Ramaciotti Foundations, 10.13039/501100001232Royal Australasian College of Physicians, and the Western Australia Department of Health.

**Research in context**

**Evidence before this study**

RCTs evaluating SMS reminders have demonstrated small to modest improvements in childhood vaccination rates and timeliness across both low-middle and high income countries. Most studies have targeted specific vaccine doses and specific subpopulations, limiting their generalisability to whole-of-population childhood vaccine programs. Whether the framing of the message content or timing of SMS reminders are important to their effectiveness is unknown.

**Added value of this study**

We evaluated a range of SMS message framings and timings via a large digitally-automated RCT embedded within the routine delivery of Australia's national immunisation program in primary care. On average, across all message strategies and vaccine schedule points, SMS reminders improved the timeliness of routine childhood vaccination by 2–11% relative to no SMS. We found evidence that neutrally framed reminders may be less effective than reminders with a positive, risk-based or social benefit framing, but no evidence that the effectiveness of SMS reminders was influenced by timing. To our knowledge, this study is the first methodological application of a fully healthcare-embedded digitally-automated clinical trial. Although the trial was capable of running independently, statistical and data quality monitoring were performed by trial staff throughout the study period to verify it was operating as intended.

**Implications of all the available evidence**

Given the high probability of effectiveness in the Australian context, provider-issued SMS reminders should be sent within one or two weeks of the scheduled date of routine childhood vaccine doses to improve vaccine timeliness. The best evidence is for messages with persuasive rather than neutral content, although superiority was not established. Key features that may have been important to the observed effectiveness are the use of software integrated with practice information systems which allowed reminders to be automatically rather than manually generated, personalisation of reminders which were issued by the child's primary care provider rather than generic reminders issued by a health department, and an opt-out rather than opt-in approach to consent to receive reminders. Given the low incremental cost of SMS, governments should fund reminders as part of the National Immunisation Program. The effectiveness of reminders in other contexts, in other age groups and for non-routine vaccines should be evaluated before being routinely implemented.

## Introduction

The sporadic resurgence of measles and other vaccine preventable diseases underscores the importance of ensuring high vaccine coverage and timeliness in children.^1^^,^^2^ Over 22 million children missed their first measles vaccine dose in 2023, an increase of 2.9 million children compared to pre-COVID-19 pandemic levels.^3^ In Australia, the proportion of children who are fully up-to-date with vaccination is high (>90%), but since the COVID-19 pandemic it has progressively fallen further short of the national target of 95% coverage.^4^ While Australia sets no official targets for the timeliness of vaccination, vaccines are typically described as occurring ‘on-time’ if delivered within a month of the scheduled due date. In some sub-populations, up to half of vaccine doses are not delivered on-time.^5^ The causes of late and incomplete vaccination are likely to include both barriers to access as well as negative or ambivalent beliefs and attitudes toward vaccination; there is evidence both determinants have been negatively affected by the COVID-19 pandemic.^6^, ^7^, ^8^, ^9^ In a recent survey of 2000 Australian parents, the self-reported factors most strongly associated with incomplete childhood vaccination were failure to prioritise vaccination over other things and challenges obtaining an appointment for vaccination.^10^

Behaviour change theories like the health belief model^11^ (HBM) have been developed to try to explain preventative health behaviours; they variously describe how beliefs and background factors like socioeconomic status interact with specific cues to influence health-related behaviour. In a previous cross-sectional survey, parents who had stronger perceptions in HBM domains of susceptibility to and severity of illness, benefits and cues to action were less likely to be hesitant in their child receiving a COVID vaccination.^12^ SMS reminders may serve as a non-coercive ‘nudge’ which, at its simplest, prompts the recipient to act upon their pre-existing intention while still allowing them to ignore or opt out of the desired behaviour.^13^ In the context of vaccination, SMS reminders may also include instructional content for cues to action for health decisions (e.g. how and when to schedule vaccination), or they can include educational or persuasive content to encourage vaccination by addressing beliefs or attitudes that impact the perceived severity of disease or benefits for prevention.

Previous studies across a number of settings have found that SMS reminders are mostly associated with small to moderate improvements in uptake and timeliness of childhood vaccination.^14^^,^^15^ Some studies have reported that educational or persuasive SMS reminders result in a small incremental benefit compared to neutral reminders that simply convey that a vaccine dose is due. A recent meta-analysis for framing in communication, not specific to SMS delivery, reported that persuasive reminder framing improved vaccine uptake compared to neutral framing (RCTs overall pooled OR 1.5; 95% CI: 1.2–2.0), with no differences observed in performance between gain-framed (positive/benefit) or loss-framed (negative) reminders.^16^ In two large RCTs of alternate framings of SMS reminders for adult influenza vaccination issued by pharmacies^17^ and GP clinics,^18^ an SMS indicating that a vaccine was being reserved for the SMS recipient was superior to no SMS. Framing vaccination as a social responsibility to protect others has been associated previously with improved willingness to vaccinate.^19^

SMS reminders have been evaluated as both pre-call reminders (before the due date) and as recall reminders (for vaccines that are overdue), with evidence that either strategy may be effective; in our recent systematic review,^14^ we identified no trials that evaluated alternative message framing and timings in a factorial design. Most studies have been conducted in high income settings and have assessed effects on specific vaccines rather than on whole vaccine schedules. Randomised trials in Australia and USA found have that SMS resulted in higher vaccine coverage at 12 months,^20^ and that parents receiving bidirectional SMS reminders were more likely to receive all vaccines compared to no SMS reminder.^21^

We aimed to estimate the effectiveness of SMS reminders (versus no SMS) on timely childhood vaccination for any eligible vaccine dose in the Australian National Immunisation Program among parents of young children in Australia. On-time vaccination is highly important in developing adequate immune responses in line with recommended dosing intervals,^22^ reduces the period that children are unprotected against disease,^23^ and is associated with improved schedule completion.^24^ Using a factorial design, we further sought to identify any effect of the content framing or the timing of the reminder (relative to the vaccine's scheduled due date). Finally, we sought to identify the single most effective combination of content framing and timing. Here we report the main analyses of the AuTOMATIC trial; further results including all pre-specified and any *post hoc* sensitivity analyses are reported in the Supplementary Material.

## Methods

### Study setting

Australia has a universal health scheme for its citizens and permanent residents. Routine childhood vaccines are funded by the Federal Government for all children through the National Immunisation Program (NIP).^25^ Most vaccines are delivered in primary care through private general (family) practice clinics, although some are delivered by public community clinics. Some children receive routine vaccines from multiple clinics. Routine NIP vaccine doses are scheduled to occur at birth and then at 2, 4, 6, 12, 18 and 48 months old. For the 2-month schedule point, vaccination is encouraged to occur as soon as possible from 6 weeks old. SMS reminders for vaccination are not routine, although some clinics issue SMS reminders for clinic appointments that have been already made. Parents whose children are overdue for routine vaccination may have certain government subsidies and benefits withheld until vaccination is complete.^26^

### Study design

AuTOMATIC was a Bayesian adaptive multi-centre randomised trial with a factorial design comparing 12 (4 × 3) SMS reminder and one control strategy (no SMS). The full study protocol has been detailed elsewhere.^27^ In brief, the trial aimed to randomise up to 10,000 parents to one of the 13 message strategies. This sample size was chosen based on feasibility and resource availability. Trial operating characteristics were evaluated through simulations which considered a range of effect sizes for the message strategies as summarised in the trial protocol and statistical analysis plan (published as a supplement to the protocol). Under the scenario where all interventions were equally effective with an odds ratio of 1.5 relative to the control, the cumulative probability of declaring effectiveness was more than 85% with the type I assertion probability controlled at less than 0.05.

The trial was conducted at 20 clinic sites in Australia participating in SmartVax, a program which uses a proprietary software system to issue SMS vaccine reminders and post-vaccination surveys to ascertain vaccine reactions. The SmartVax coordination team invited clinics already issuing post-vaccine surveys (but not vaccine reminders) to take part in the trial. There was additional promotion via local networks of primary health care providers.

**Fig. 1:**
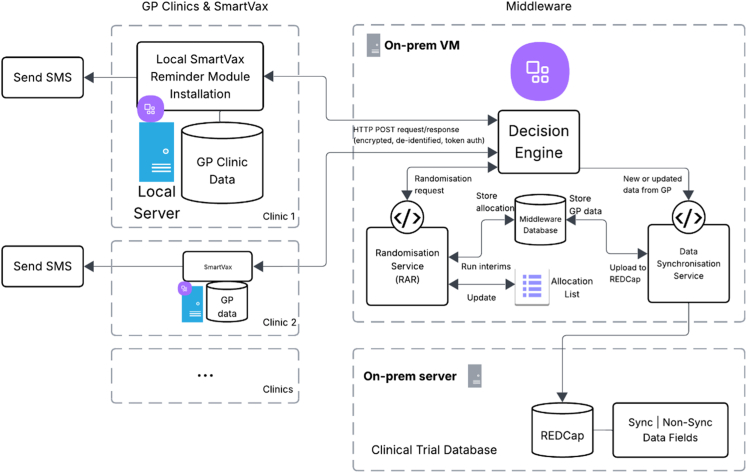
**AuTOMATIC digital infrastructure**.

The SmartVax software was being used in approximately 400 primary care clinics across Australia at the time of the study.^28^ Bespoke digital infrastructure was developed to implement and automate trial procedures (Fig. 1). A middleware application was used to store coded procedures and relay de-identified parent/child data, random allocations and SMS/vaccination event data between SmartVax and a secure study database (REDCap). The middleware application also coordinated interim analyses, updated allocation probabilities and produced the randomisation schedule for the next cohort of participants. Study staff regularly monitored trial progress, adaptations and automated alerts for data upload errors or system bugs. All analyses were monitored by two independent statistical monitors.

### Participants

Under a waiver of consent, we randomised all parents of singleton children who were registered at participating clinics, and who were either <2 months old, or 2 months to <4 years old and having a record of previous vaccination at the clinic. We excluded those who had previously opted out of SMS communication with the clinic, and those without a name or without a valid date of birth or mobile phone number recorded in the clinic's electronic practice information system.

### Intervention

**Fig. 2:**
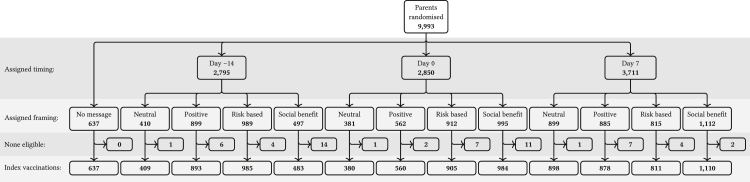
**CONSORT diagram**.

The intervention was an SMS reminder sent to parents of eligible children, containing a specific combination of one of four possible content framings and one of three timings in relation to the vaccine's scheduled due date, or SMS reminder. SMS content (Supplementary Material Figure S1*)* comprised a personalised reminder from the issuing clinic (hereafter the ‘source’ clinic) and addressed to the parent of the eligible child. The four alternative framings were: a neutral reminder simply conveying that a vaccine was due; a positively-framed reminder which promoted the personal benefits of vaccination; a risk-based reminder that warned of the danger to the child of delaying vaccination; and a social benefit framed reminder which promoted the benefit to the community as well as the individual child (Fig. 2). The SMS content options were co-developed with a 12-member community reference group and then selected via an online survey of 89 parents across Australia. The survey requested parents to provide feedback on appropriateness and to choose their preferred message in each of the four content domains, with mean ranks used to select the most preferred message in each content domain for the trial. SMS reminders could be issued at one of three randomly assigned timing options: either 14 days before vaccination was scheduled to occur (the due date), on the due date, or 7 days after the due date. SMS reminders were withheld on occasions where the scheduled vaccine was recorded as having been administered at the source clinic prior to the scheduled message date or where children had not met the minimum window between eligible vaccine doses (i.e. 4 weeks). Each SMS instructed parents how they could opt out of future reminders.

### Trial procedures

Eligibility screening was automated using SmartVax software installed on each clinic's local server. The software interrogated the practice management system each day of the trial enrolment period to identify any children meeting the eligibility criteria and due for a scheduled vaccine dose within the following 28 days. An anonymised child-parent identifier was created from a complex hash of the child's name, date of birth and parent's mobile phone number. This allowed us to identify any child-parent dyads registered with multiple participating clinics, and ensured parents received the same assignment for all scheduled vaccines for that child and any of their siblings. Our randomisation logic (described below) checked whether any unique child or parent ID combinations had been previously assigned to any specific group allocation and returned that group assignment to any siblings associated with the parent ID.

Each day, the software system automatically extracted updates to the following data from the source clinic electronic information system for each scheduled vaccine for each participating child: the child-parent identifier, the source clinic identifier, vaccine schedule point (age 2, 4, 6, 12, 18, and 48 months), the due date, age of child, whether an SMS was sent, and any new vaccine doses captured in the previous 24 h. Data was uploaded to the middleware application and then pushed to the trial database for storage.

### Randomisation and masking

Randomisation lists were generated using an R script stored in the middleware's decision engine. The middleware application randomly assigned parents to one of the 12 SMS reminder arms or the control arm based on an allocation list which was generated using a mass-weighted urn design.^29^ Initially, the allocation probabilities were 1/5 to the control arm and 1/15 to each of the 12 SMS reminder arms, resulting in a 1/5 allocation to each of the four reminder content framings, and 4/15 to each of the three reminder timings. Allocation to the no SMS reminder arm was to remain fixed at 1/5 for the duration of the trial unless the arm was discontinued for inferiority per pre-specified criteria described below. Allocation ratios to the SMS reminder arms were updated after each interim analysis using Bayesian response-adaptive randomisation (RAR). RAR is a method of randomisation that allows the trials allocation probabilities to be revised considering accrued outcomes with an aim to achieve some trial objective. Here, RAR was used to favour assignment to better performing arms, particularly those with less precise estimates.^30^ Following pre-scheduled interim analyses the probability of assigning new parents to a reminder arm was updated using the primary analysis model with weights proportional to the square root of the probability that the reminder arm was best, the variance on conditional log-odds of vaccination for that arm, and the inverse of the current sample size of that arm.

While aware of any SMS received, parents were not informed of the study. The arm allocations were concealed from clinic staff and all investigators except the trial statistician who performed the interim and final analyses. No steps were taken to avoid parents unblinding clinic staff to their assignment.

### Outcome measures

The primary endpoint was on-time vaccination, defined as receipt of a scheduled vaccine at the source clinic within the period from 14 days prior to its scheduled due date to 28 days afterwards. It was not possible to determine why a record of vaccination receipt may have been absent, therefore any participant with no record of vaccination receipt was considered not to have met the primary outcome, meaning there was no missing outcome data A secondary endpoint was the day of vaccine receipt relative to its scheduled due date (reported in Supplementary Material).

### Statistical analysis

Analyses were pre-specified in an analysis plan, published in the Supplementary Material to the trial protocol.^27^ An independent statistical monitoring committee (ISMC) provided oversight to ensure analyses and decision rules were implemented as pre-specified. Analyses were performed with parents grouped according to the assigned intervention and all included (i.e. intent-to-treat). Receipt of sent messages could not be confirmed, so no per-protocol analyses were performed. We excluded from the analysis all randomised vaccine occasions where there was a record of the vaccine dose having been administered more than 14 days before its scheduled due date (i.e. prior to when any SMS reminders could be issued; we included all occasions where the vaccine was administered from 14 days before its schedule date, even if this occurred before an SMS was issued. A small number of clinic server outages that prevented SMS from being sent to randomised recipients were documented in the protocol deviation log and monitored by the ISMC. A parent's index vaccine occasion was taken to be the primary unit of analysis and was defined as the first SMS-eligible vaccine occasion for a child of that parent following randomisation; there was therefore only one index vaccine occasion per parent even though each parent may have multiple children, and each child may have multiple vaccine occasions which were eligible for an SMS reminder. Secondary analyses included data for both index and non-index vaccine occasions, i.e. all SMS-eligible scheduled vaccine occasions for all children of each participating parent.

The primary analysis of on-time vaccination was performed on data for the index vaccine occasion for each parent and used a Bayesian logistic regression model to estimate the conditional odds of vaccination by day 28 for each of the 12 message combinations and no SMS. The full model contained parameters for the effects of each message timing and framing and the effects of timing-by-framing interactions while adjusting for infant age (2, 4, 6, 12, 18, or 48 months), source clinic (random effect), and calendar time of the due date (4-week intervals). Reduced models, each adjusting for the same covariates, were also estimated with: no interaction between reminder timing and framing, only framing effects, only timing effects, and only a shared effect pooling all types of message reminders. The models were fit using Markov chain Monte Carlo with 10,000 draws from the joint posterior.

Secondary analyses (see Supplementary Material) included data for all SMS-eligible vaccine occasions for all children of each parent using Bayesian logistic regression models as previously described with the addition of parent-specific random effects. We assessed heterogeneity of treatment effects with respect to the vaccine schedule point (child age); in each model, the effect of SMS reminders was modelled as specific for each vaccine schedule point rather than shared across all schedule points.

To aid interpretation of the size of the effect of the SMS reminders, marginal differences in the proportion vaccinated on time were calculated under all models for the most recent calendar time and a “typical” source clinic (and parent as appropriate) defined as the random effect parameter being equal to zero, with a simple (equal-weighted) average taken over all vaccine schedule points (or instead reported as schedule point-specific differences).

The study data were analysed at pre-specified interim analyses and the results of the analyses used to update target allocation ratios to the trial arms. The first interim analysis occurred after 1500 index scheduled vaccine occasions completed the 28 days of follow-up required to ascertain the primary outcome so that at least 100 parents were assigned to each intervention. Subsequent interim analyses occurred after every additional 500 index vaccine occasions. The control arm was planned to be discontinued if either the average reminder effect, or any one of the 12 individual reminder types, had high probability of superiority compared to no SMS with respect to on-time vaccination. Following the 3rd interim analysis, the primary analysis showed a high posterior probability of superiority of at least one SMS reminder type over no SMS, and for the pooled effect across all reminder types compared to no SMS. Based on the results of this interim analysis, the no SMS arm was discontinued from the trial and all subsequently enrolled parents were assigned to one of the 12 SMS reminder arms. Implementation of this rule was reviewed and acknowledged by the independent statistical monitors. At trial completion, secondary analyses were performed in which the sample was restricted to only those parents who were randomised prior to completion of the 3rd interim analysis (i.e. only parents who could have been assigned to the no SMS control arm).

Analyses were conducted in R (4.4.1).^31^ Bayesian models were estimated using Stan (2.35.0)^32^ via cmdstanr (0.8.1).^33^ Full model specifications are reported in the Supplementary Material.

### Ethics approval

A waiver of the requirement for participant consent was requested and approved on the basis that disclosing the trial objectives would undermine its integrity and because any risk to participants was likely to be low with the use of deidentified data. The potential benefits of parents receiving reminders from their existing practice and achieving on-time vaccination outweighed the low-negligible risk of parents interpreting reminders as intrusive; information to opt-out was provided in the messages to further reduce this a minor inconvenience and opt-out rates were monitored by the study team. Approval of the protocol was granted by the University of Western Australia Human Research Ethics Committee (Ref: 2019/RA/4/1/8810|2024/ET000011).

### Role of the funding source

The funder of the study had no role in study design, data collection, data analysis, data interpretation, or writing of the report.

## Results

Between January 14, 2021, and February 26, 2024, 9993 parents from 20 clinics were randomised (Fig. 2). Randomisation was stopped just prior to reaching the maximum number of parents. At least one clinic was located in each of the seven Australian states or territories, except for South Australia (Supplementary Table S1). Four clinics were in rural local government areas. Two clinics were located in local government areas in the lowest quintile (disadvantage) and six in the highest quintile (advantage) using the index of relative socioeconomic advantage and disadvantage (IRSAD) as assessed by the Australian Bureau of Statistics.^34^

A total of 11,541 children of these parents had at least one scheduled vaccine occasion between February 04, 2021 and March 25, 2024, with 22,865 total scheduled vaccine occasions. Of the participating parents, 8559 (86%) had a scheduled vaccine occasion for one child, 1327 (13%) for two children, 100 (1%) for three, and 7 for four. After a first SMS reminder, one parent opted out of receiving further reminders via their clinic reception team.

Of the 22,865 scheduled vaccine occasions, 1366 (6%) were not eligible to receive a SMS reminder due to early vaccination (more than 14 days before the due date). These were mostly for the 2-month schedule point which accounted for 1310 (96%) of such occasions. From the remaining 21,499 SMS-eligible vaccine occasions, there were 9933 index vaccine occasions. Of these, 637 (6.4%) parents were assigned to no SMS, and the number of parents assigned to each SMS reminder combination ranged from 380 (3.8%) to 1110 (11.2%) (Table 1).

**Table 1:** Number of index vaccinations assigned to each reminder combination, the number (proportion) vaccinated on time (by day 28), and the number (proportion) vaccinated within intervals with endpoints defined by reminder timings.

Timing	Framing	Index vaccinations	Vaccinated on time	Vaccination timing (days)		
				(−14, 0)	(0, 7)	(7, 28)
None	None	637	286 (0.45)	31 (0.05)	118 (0.19)	137 (0.22)
Day −14	Neutral	409	168 (0.41)	21 (0.05)	73 (0.18)	74 (0.18)
	Personal benefit	893	420 (0.47)	63 (0.07)	204 (0.23)	153 (0.17)
	Personal risk	985	438 (0.44)	69 (0.07)	202 (0.21)	167 (0.17)
	Pro-social	483	209 (0.43)	37 (0.08)	95 (0.20)	77 (0.16)
Day 0	Neutral	380	153 (0.40)	16 (0.04)	68 (0.18)	69 (0.18)
	Personal benefit	560	246 (0.44)	36 (0.06)	108 (0.19)	102 (0.18)
	Personal risk	905	400 (0.44)	61 (0.07)	168 (0.19)	171 (0.19)
	Pro-social	984	468 (0.48)	68 (0.07)	198 (0.20)	202 (0.21)
Day 7	Neutral	898	389 (0.43)	59 (0.07)	132 (0.15)	198 (0.22)
	Personal benefit	878	409 (0.47)	59 (0.07)	149 (0.17)	201 (0.23)
	Personal risk	811	363 (0.45)	43 (0.05)	142 (0.18)	178 (0.22)
	Pro-social	1110	489 (0.44)	73 (0.07)	185 (0.17)	231 (0.21)

The proportion of parents assigned to each SMS reminder combination following each interim analysis, and the distribution of ages, clinics, and cohorts, amongst reminder combinations, are reported in the Supplementary Material (Supplementary Figure S2 and Supplementary Tables S2 & S3).

Overall, the proportion of vaccine occasions resulting in on-time vaccination (within 28-days of the due date) was highest at the 4-month schedule point and gradually declined for later schedule points (Supplementary Figure S3). For the 2-month schedule point, most vaccines were given prior to the due date.

**Fig. 3:**
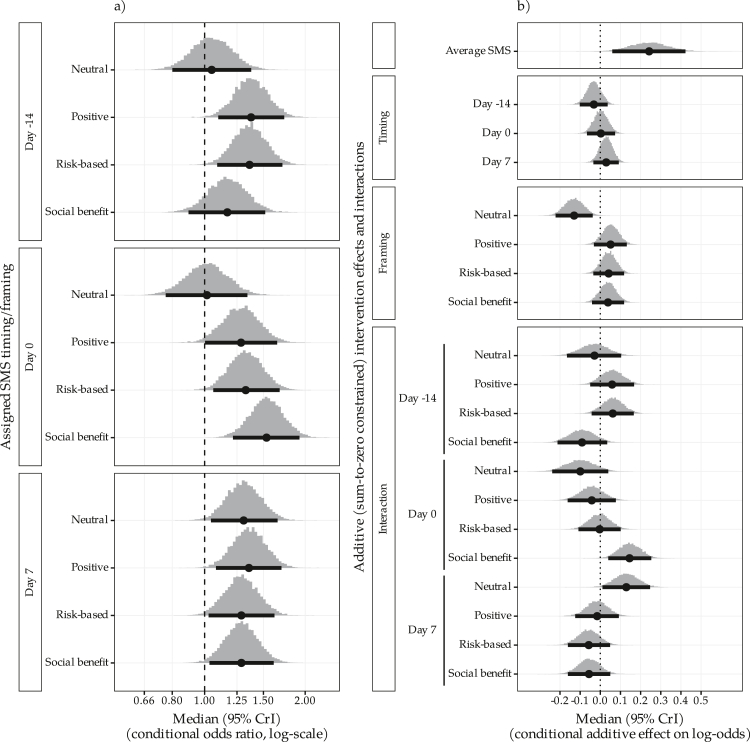
**a) Posterior density and median (95% CrI) for conditional odds ratios of each reminder combination versus no reminder, b) additive main and interaction effects on conditional log-odds of on-time vaccination for reminder components under the full model for SMS-eligible index vaccinations**.

In the primary analysis of index vaccine occasions, the adjusted posterior odds ratios (aOR) of on-time vaccination for each SMS reminder combination compared to no SMS ranged from a median of 1.02, [95% credible interval (0.76, 1.34); Pr (>1) = 0.55] to 1.53, [(1.22, 1.92); Pr (>1) >0.99] (Fig. 3 and Table 2 and Supplementary Figure S4). Differences between message timings were on average (across framings) unlikely to be large with 7 days after versus 14 days before the due date, aOR 1.06 (0.95, 1.19) and message framings had on average (across timings) similar performance except for the neutral framing. For example, positive versus neutral aOR 1.20 (1.04, 1.4). Under a homogenous reminder effect model, the pooled posterior aOR for a message versus no message was 1.29 [(1.06, 1.55); Pr (>1) = 0.99]. For a “typical” site with equal weighting across vaccine schedule points, this equated to an estimated marginal absolute increase in the proportion vaccinated on time of 6% (2%–11%) (Supplementary Table S4). When data on all SMS-eligible vaccine occasions were considered, the estimated reminder specific aORs from the full model for on-time vaccination ranged from 0.87 [(0.65, 1.16); 0.17] to 1.42 [(1.12, 1.82); >0.99] (Supplementary Table S5 and Supplementary Figure S5). The shared reminder effect model aOR was 1.24 [(1.03, 1.49); 0.99], which for a “typical” site and parent, equated to a marginal increase in proportion vaccinated on time of 5% (1%–10%) (Supplementary Table S6).

**Table 2:** Posterior summary of message effect conditional odds ratios relative to no message for day 28 vaccination status amongst SMS eligible index vaccinations, by the assumed model.

	Full (primary)		No interaction		Timing only		Framing only		Shared	
	Median (95% CrI)	Pr (>1)	Median (95% CrI)	Pr (>1)	Median (95% CrI)	Pr (>1)	Median (95% CrI)	Pr (>1)	Median (95% CrI)	Pr (>1)
**All timings**										
All framings									1.29 (1.06, 1.55)	0.99
Neutral							1.15 (0.93, 1.43)	0.90		
Positive							1.33 (1.08, 1.62)	1.00		
Risk-based							1.30 (1.06, 1.60)	0.99		
Social benefit							1.33 (1.08, 1.62)	1.00		
**Day 14**										
All framings					1.26 (1.03, 1.53)	0.99				
Neutral	1.05 (0.80, 1.38)	0.64	1.12 (0.90, 1.40)	0.85						
Positive	1.38 (1.10, 1.73)	1.00	1.30 (1.06, 1.60)	0.99						
Risk-based	1.36 (1.09, 1.71)	1.00	1.27 (1.04, 1.57)	0.99						
Social benefit	1.17 (0.90, 1.52)	0.88	1.29 (1.04, 1.60)	0.99						
**Day 0**										
All framings					1.31 (1.08, 1.60)	1.00				
Neutral	1.02 (0.76, 1.34)	0.55	1.17 (0.94, 1.46)	0.92						
Positive	1.29 (1.00, 1.65)	0.98	1.35 (1.09, 1.67)	1.00						
Risk-based	1.33 (1.06, 1.68)	0.99	1.33 (1.08, 1.63)	1.00						
Social benefit	1.53 (1.22, 1.92)	1.00	1.34 (1.09, 1.65)	1.00						
**Day 7**										
All framings					1.29 (1.06, 1.56)	0.99				
Neutral	1.31 (1.04, 1.65)	0.99	1.16 (0.94, 1.43)	0.92						
Positive	1.36 (1.08, 1.70)	1.00	1.34 (1.09, 1.65)	1.00						
Risk-based	1.29 (1.03, 1.62)	0.99	1.32 (1.07, 1.62)	0.99						
Social benefit	1.29 (1.03, 1.61)	0.99	1.33 (1.09, 1.64)	1.00						

When restricting the analysis to only include data for parents who were randomised before discontinuation of the no SMS control arm, the overall results were like those obtained for the full cohort. The saturated model aORs ranged from 0.94 [(0.64, 1.38); 0.39] to 1.69 [(1.19, 2.43); >0.99] with a pooled reminder effect of 1.32 [(1.07, 1.62); 0.99] (Supplementary Tables S7 and S8 and Supplementary Figures S6 and S7).

In analyses of the heterogeneity of reminder effects with respect to schedule point (child age) under the pooled reminder effect model for all SMS-eligible vaccine occasions, the aORs of on-time vaccination for any reminder compared to no reminder were highest for the 18-month schedule point [1.58, (1.18, 2.09; >0.99] and 48-month schedule point [1.45, (1.07, 2.00); 0.99] with marked uncertainty about any beneficial reminder effect at the earlier vaccine schedule points (Supplementary Tables S9 and S10). More detailed reporting on posterior summaries for all secondary and post-hoc analyses are provided in Supplementary Tables S11 and S12 and Supplementary Figures S4–S12.

## Discussion

SMS reminders were, overall, associated with a modest but meaningful absolute improvement of 6% (2–11%) in on-time vaccination of children compared to no SMS reminder. At an assumed incremental cost of issuing SMS reminders of $AUD0.20 per SMS, a 6% improvement would equate to a cost-benefit ratio as approximately $4 per additional on-time vaccination in the study setting.

The alternatively framed persuasive reminders were similarly effective compared to no SMS, but neutral reminders were associated with no improvement in timeliness for two of the three message timing options. Improved effectiveness of persuasive over neutral framings is consistent with the findings of other trials.^14^ There is vast literature investigating the importance of framing effects in general health communication^35^ and vaccination,^36^ primarily assessing the impact of framing on beliefs or intent, rather than actual behaviour or clinical outcomes. Much of this literature contrasts gain-framed and loss-framed messaging, arising from the prospect theory of Tversky and Khaneman^37^ that posits that people may be more strongly risk-aversive than gain-seeking depending on context. While appeals to social benefit appeared to improve support of administering some vaccines to adults,^38^ it did not appear to improve parental support of vaccination for their own children against either measles-mumps-rubella^39^ or COVID-19.^40^ There was no clear difference observed in the effectiveness of SMS reminders by their timing of issue. From a programmatic perspective, only issuing recall SMS reminders after children have past their due date by a week would entail fewer reminders and lower cost and could result in similar on-time vaccination compared to universal pre-call reminders.

The SMS reminders were personalised and sent from a primary care clinic with whom parents were registered and therefore had an existing relationship. In Australia, general practitioners are primarily responsible for delivering routine childhood vaccines and are also widely trusted as a source for vaccine information^41^; it is unclear whether reminders sent anonymously or from other sources (e.g. government) would have had similar acceptance by parents or response rates. Only a single request to opt out was received from over 20,000 unsolicited SMS reminders issued, suggesting that even if not always acted upon, the reminders were tolerated well by parents.

The trial took place in a middle-to high-income population with high vaccine coverage relative to international standards. In 2023, the overall vaccine coverage in Australia was 93% for 12-month-olds and for 5-year-olds. It is reasonable to expect that the absolute benefit of reminders may be greater in settings with lower baseline on-time vaccination rates, although improving vaccine timeliness in lower resource settings may be more dependent on improvements in access.^42^ Our results are therefore not necessarily generalisable to other contexts. We did not specifically target reminders to Australian First Nations or other culturally or linguistically diverse families and nor did we capture data on ethnicity. While our SMS wording was co-developed with a community reference group and validated via a parent survey, these were primarily English-speaking parents and reminders may need to be co-developed and evaluated in diverse communities to ensure broad appropriateness and effectiveness.

The trial commenced in 2021 while population-wide roll out of COVID-19 vaccines was occurring. According to the Australian Immunisation Register, over the four years to the end of 2023, on-time vaccination for non-Indigenous Australian children declined to 84% for the 4-month diphtheria-tetanus-pertussis vaccine dose and 67% for the 12-month measles-mumps-rubella vaccine dose.^43^ On-time coverage was approximately 10 percentage points lower for First Nations children at both schedule points.^43^ There is evidence that ease of access to routine primary care and community sentiments toward vaccination deteriorated in Australia over this time period,^9^^,^^44^ and we observed that vaccine timeliness declined in the later time epochs of the trial (*see supplementary material*). It is therefore possible that the observed SMS reminder effects are relatively specific to the era of the trial.

While automation of trial processes is being increasingly explored for specific trial processes like eligibility screening^45^ and data extraction,^46^ to our knowledge this is one of the most fully automated trials ever implemented. Screening, randomisation, issuing of assigned SMS reminders at the assigned time, ascertainment of the outcome (date and time of vaccination), and to a large extent, the scheduled interim analyses, were automated. The only direct involvement of participants was with clinics via the SMS reminders and the encounters for vaccination. After agreeing to take part and ensuring installation of the necessary software, no additional study-related procedures were required of the clinics. A trial statistician and two independent statisticians monitored to ensure pre-specified analyses and trial rules for stopping and adaptations were implemented per the analysis plan. The technical team occasionally needed to resolve software outages and intervene when clinic servers went ‘offline’.

High levels of automation enabled almost complete enrolment of the eligible study population, mitigating the risk of selection bias and therefore improving the generalisability of the results from the trial to the target population of all Australian families with vaccine-eligible children. This, in turn, was possible because of a waiver of the need for opt-in consent. This prevented the inadvertent selection of parents with a positive disposition towards vaccination and the distortion that is likely to have occurred if parents had foreknowledge of the study and its objectives.

High levels of automation allowed us to implement the trial with a total budget of approximately $AUD210,000, equating to just $21 per randomised parent. While automation can help improve cost-efficiency, the time and resources required to design and build the infrastructure for such a trial can be substantial. In the case of this trial, it took approximately 4 years from the awarding of funding to the enrolment of the first participant, and most of the budget was expended in this period. This highly automated approach is likely to have wide applicability for evaluating other areas of healthcare, especially for low-risk digital interventions that can be implemented, evaluated and optimised over time within a learning health system model of care.

A main limitation of the trial is that we were unable to ascertain vaccine receipt outside of the source clinics. Because Australian parents are free to choose the vaccine provider for their child, it is possible that the reminders diverted vaccine delivery toward the source clinics from elsewhere, with little or no overall increase in on-time vaccination. As a further limitation, we were unable to assess the long-term impact of reminders on completion of all timepoints of the vaccine schedule. Elsewhere, on-time vaccination has been associated with an increased likelihood of completing multi-dose vaccine series.^47^, ^48^, ^49^ Still, it is possible that SMS reminders brought forward vaccine doses that would have eventually occurred regardless, so we cannot claim an effect of reminders on schedule completion. Arguably, achieving on-time vaccination is an important goal in its own right as early childhood is a time of increased susceptibility to many vaccine-preventable diseases. Although we can't assess how representative the 20 clinics involved in the study were compared to the broader Australian population, the centres geographically fell mostly into middle to high socioeconomic catchment areas, which may impact the generalisability of our findings. The waiver of consent approach meant that we were unable to capture individual parent and child demographic characteristics, which limited our ability to capture additional data that might have helped interpretation of the results. In particular, we were unable to assess whether effectiveness was modified by sociodemographic factors or the effect of reminders on parental perceptions, beliefs or attitudes towards vaccination. While no steps were taken to prevent staff from being unblinded, this information was difficult for staff to access via the SmartVax software. For a staff member to obtain information on group allocation, they would need to find and log in to the application installed on the clinic server which was not required for daily administration tasks and locate the logs of sent SMS reminders. We cannot envisage a scenario where staff were motivated to alter a requested vaccination based on a suspicion about the participant's group allocation. Clinic representatives were asked to not alter their usual processes during onboarding of software.

We used sequential Bayesian analyses with response adaptive randomisation to maximise trial efficiency. In multi-arm trial settings where a truly best performing arm exists, such designs can be efficient for identifying that arm compared to conventional fixed trial designs.^50^ While a single best performing arm was not identified in our trial, early discontinuation of the no SMS arm for inferiority allowed us to minimise assignment to this arm. Any gain in efficiency, however, needs to be weighed against the increased dependence on model assumptions. The responses observed across the SMS reminder arms are not directly comparable without first adjusting for possible effects of calendar time (time epoch) and differences in clinic distribution across the arms. Early discontinuation of the no SMS arm means that comparisons against control are particularly reliant on these adjustments. Results from sensitivity analyses restricted to parents randomised before the no SMS arm was discontinued were largely consistent with those obtained for the primary model using data from all parents, providing some assurance that the model-based adjustments for the primary analysis were adequate. Similarly, we observed a low response rate for some of the neutral SMS reminder arms at the first analysis, causing these arms to receive relatively low allocation early in the trial when on-time vaccination appeared highest (see Supplementary Material). We note that this will have biased against the neutral SMS reminders if our epoch-based adjustment for time-related confounding of vaccine effects was inadequate. To reduce the variance in estimates and RAR at early interim analyses, it may have been better to delay the first interim analysis until more participants had been assigned or to use more informative priors on differences between message effects, for example a preference for zero interaction between timing and framing.

In conclusion, we found evidence that SMS reminders are effective for achieving modest but important gains in on-time vaccination for young children. There was no definitive evidence that any specific SMS framing or timing combination was likely to be better than all others, but there was evidence that persuasive reminders may be more effective than neutral reminders. Future research should assess the effectiveness of SMS reminders in special at-risk populations, including Australian First Nations families who have both reduced vaccine timeliness and high disease burden, for whom barriers to access may play a greater role, and for whom reminder content and timing may need to be customised to ensure appropriateness and for optimal effect.

## Contributors

All authors meet criteria for ICMJE recommendations and had access to all the data in the study and accept the responsibility for publication. GC was involved in the design of the trial and SMS intervention content, infrastructure design and implementation, interpreted study results and analyses performed by JT. GC and JT co-wrote the first manuscript draft. JT was involved in the study design and wrote the automated R script for study implementation. CW, JM, CCB, AL and IP contributed to study design and review of the manuscript. CH and KA contributed to design of the text message content options and review of the manuscript. MJ provided quality assurance and assistance in the interpretation of the statistical analyses, and review of the manuscript. GB and GB assisted in the design, development and management of the middleware application that enabled the infrastructure for the trial and review of the manuscript. TS contributed to all aspects of the study including providing oversight to design, implementation, authorship and final approval of the manuscript. GC, JT, MJ & TS have accessed and verified the underlying data in this manuscript.

## Data sharing statement

Population level summaries and aggregated data can be accessible upon request by contacting the corresponding author. Data requests for the full AuTOMATIC dataset will be referred to the original HREC that approved the waiver of consent to ensure the proposed use of the data is in the public interest and meets requirements for continued use of the deidentified data as a waiver of consent.

## Declaration of interests

All authors declare no conflicts of interest unless otherwise specified. AL and IP are owners of SmartVax and receive funding from Australian Government to support the program. They were involved in the design and interpretation of the results but had no role in the statistical analysis. KA declares research funded by the Government of Western Australia and the Medical Research Future Fund of the Australian Government. TS declares an Investigator Grant from the Australian Medical Research Future Fund (MRF1195153).

## Appendix B Supplementary data

Supplementary File

Study Protocol
