# Supplementary material for: The AuTOMATIC trial: a multicentre digitally-automated, Bayesian, adaptive, parallel, factorial randomised controlled trial of SMS reminders for childhood vaccination
Source: Lancet Reg Health West Pac. 2026 Feb 11;67:101804. doi: 10.1016/j.lanwpc.2026.101804 (PMC12958076; doi:10.1016/j.lanwpc.2026.101804)
Supplement: Supplementary File [file mmc1.pdf]

Supplemental Information

The AUTOMATIC trial: SMS reminders improve childhood vaccination in a digitally-automated, Bayesian, adaptive, multicentre, parallel, randomised controlled trial.

Table of contents

1 SMS Examples **3**

2 Statistical Models **4**
2.1 Day 28 vaccination 4
2.2 Day of vaccination 5

3 Baseline and Descriptive Statistics **6**

4 Statistical Analysis - Day 28 Vaccination Results **11**
4.1 SMS eligible index vaccinations 11
4.2 All SMS eligible vaccinations 13
4.3 SMS eligible index vaccinations amongst parents randomised contemporaneous with control . . 16
4.4 All SMS eligible vaccinations amongst parents randomised contemporaneous with control . . . 18
4.5 Age-specific effects 20

5 Statistical Analysis - Day of Vaccination Results **21**
5.1 Age-specific time-varying effect of message 24

List of Figures

S1 SMS examples. XX/XX/XX was populated by a date and call XXXX XXXX was populated by the clinic phone number. 3
S2 Proportion of newly encountered parents allocated to each message type by allocation cohort. The start date for each set of allocation proportions is given along with the total number of parents randomised. Vertical lines indicate target proportions under equal allocation without control group (1/12). Highlighted panels indicate control arm availability. 7
S3 Cumulative proportion of vaccinations administered and empirical hazard by age for SMS eligible index vaccinations, and all SMS eligible vaccinations. 10
S4 Posterior summaries of age, clinic, and epoch odds ratios for day 28 vaccination status amongst SMS eligible index vaccinations, by assumed model. Points – median, rectangles – 80% CrI, lines – 95% CrI. 12
S5 Posterior summaries of age, clinic, and epoch odds ratios for day 28 vaccination status amongst all SMS eligible vaccinations, by assumed model. Points – median, rectangles – 80% CrI, lines – 95% CrI. 15
S6 Posterior summaries of age, clinic, and epoch odds ratios for day 28 vaccination status amongst index SMS eligible vaccinations for parents randomised while control arm available, by assumed model. Points – median, rectangles – 80% CrI, lines – 95% CrI. 17
S7 Posterior summaries of age, clinic, and epoch odds ratios for day 28 vaccination status amongst all SMS eligible vaccinations for parents randomised while control arm available, by assumed model. Points – median, rectangles – 80% CrI, lines – 95% CrI. 19

S8 Estimated baseline hazard (no message) under the full model by scheduled vaccination age, typical clinic. . . . 22
S9 Posterior difference in probability of vaccination by day relative to scheduled due date for a "typical" clinic (zero-effect) and simple average across all vaccination ages under the full model with constant hazard ratios. Solid black line indicates posterior median and filled bands credible intervals. . . . 22
S10 Estimated vaccination age, clinic, and epoch continuation ratios for day of vaccination amongst SMS eligible index vaccinations, by assumed model. Points – median, rectangles – 80% CrI, lines – 95% CrI. . . . 23
S11 Posterior difference in cumulative incidence of vaccination by day relative to scheduled due date under a "timing-only" model with time-varying hazard ratios for a "typical" clinic (zero-effect). by vaccination age. Solid black line indicates posterior median and filled bands credible intervals. 24
S12 Posterior difference in cumulative incidence of vaccination by day relative to scheduled due date under a "timing-only" model with time-varying hazard ratios for a "typical" clinic (zero-effect) and simple average across all vaccination ages. Solid black line indicates posterior median and filled bands credible intervals. . . . 25

List of Tables

S1 Summary of participating clinics. . . . 6
S2 Distribution of vaccination ages, participating clinic, and parent allocation cohort, by assigned message type, for SMS eligible index vaccinations. . . . 8
S3 Distribution of vaccination ages, participating clinic, and parent allocation cohort, by assigned message type, for all SMS eligible vaccinations. . . . 9
S4 Posterior summary of message effect difference in proportion vaccinated by day 28 relative to no message amongst SMS eligible index vaccinations, by assumed model. Proportions are calculated with respect to a "typical" clinic (zero-effect), and a simple (unweighted) average over all vaccination ages. . . . 11
S5 Posterior summary of message effect odds ratios relative to no message for day 28 vaccination status amongst all SMS eligible vaccinations, by assumed model. . . . 13
S6 Posterior summary of message effect difference in proportion vaccinated by day 28 relative to no message amongst all SMS eligible vaccinations, by assumed model. Proportions are calculated with respect to a "typical" clinic and parent (zero-effect), and a simple (unweighted) average over all vaccination ages. . . . 14
S7 Posterior summary of message effect odds ratios relative to no message for day 28 vaccination status amongst index SMS eligible vaccinations for parents randomised while control arm available, by assumed model. . . . 16
S8 Posterior summary of message effect odds ratios relative to no message for day 28 vaccination status amongst all SMS eligible vaccinations for parents randomised while control arm available, by assumed model. . . . 18
S9 Posterior summary (median and 95% credible interval) of age-specific odds ratio and difference in proportion vaccinated by day 28 (for typical clinic) of message relative to no message under shared effect model, for index vaccinations and all SMS eligible vaccinations. . . . 20
S10 Posterior summary (median and 95% credible interval) of age-specific odds ratio and difference in proportion vaccinated by day 28 (for typical clinic) of message type relative to no message under timing-only effect model, for index vaccinations and all SMS eligible vaccinations. . . . 20
S11 Estimated message continuation ratios relative to no message for day of vaccination amongst SMS eligible index vaccinations. . . . 21

1 SMS Examples

Content	Example text: 14 days before scheduled vaccine date	Example text: scheduled vaccine date	Example text: 7 days after scheduled vaccine date
Neutral	"[CLINIC NAME]: [Child's name] is due for a X-month vaccination on XX/XX/XX. To book or opt-out of SMS, call XXXX XXXX. Replies are not monitored, pls ignore SMS if you have an appt"	"[CLINIC NAME]: [Child's name] is due for a X-month vaccination today. To book or opt-out of SMS, call XXXX XXXX. Replies are not monitored, pls ignore SMS if you have an appt"	"[CLINIC NAME]: [Child's name] is overdue for a X-month vaccination. To book or opt-out of SMS, call XXXX XXXX. Replies are not monitored, pls ignore SMS if you have an appt"
Positive	"[CLINIC NAME]: [Child's name] is due for a vaccination on XX/XX/XX. Vaccinating on time gives [Child's name] the best defence against infectious diseases. To book or opt-out of SMS, call XXXX XXXX. Replies are not monitored, pls ignore SMS if you have an appt"	"[CLINIC NAME]: [Child's name] is due for a vaccination today. Vaccinating on time gives [Child's name] the best defence against infectious diseases. To book or opt-out of SMS, call XXXX XXXX. Replies are not monitored, pls ignore SMS if you have an appt"	"[CLINIC NAME]: [Child's name] is overdue for a vaccination. Vaccinating on time gives [Child's name] the best defence against infectious diseases. To book or opt-out of SMS, call XXXX XXXX. Replies are not monitored, pls ignore SMS if you have an appt"
Negative	"[CLINIC NAME]: [Child's name] is due for a vaccination on XX/XX/XX. Delaying a child's vaccination can put them and other children at risk. To book or opt-out of SMS, call XXXX XXXX. Replies are not monitored, pls ignore SMS if you have an appt"	"[CLINIC NAME]: [Child's name] is due for a vaccination today. Delaying a child's vaccination can put them and other children at risk. To book or opt-out of SMS, call XXXX XXXX. Replies are not monitored, pls ignore SMS if you have an appt"	"[CLINIC NAME]: [Child's name] is overdue for a vaccination. Delaying a child's vaccination can put them and other children at risk. To book or opt-out of SMS, call XXXX XXXX. Replies are not monitored, pls ignore SMS if you have an appt"
Social benefit	"[CLINIC NAME]: [Child's name] is due for a vaccination on XX/XX/XX. Vaccinating on time protects [Child's name] and other children who are too young to be vaccinated. To book or opt-out of SMS, call XXXX XXXX. Replies are not monitored, pls ignore SMS if you have an appt"	"[CLINIC NAME]: [Child's name] is due for a vaccination today. Vaccinating on time protects [Child's name] and other children who are too young to be vaccinated. To book or opt-out of SMS, call XXXX XXXX. Replies are not monitored, pls ignore SMS if you have an appt"	"[CLINIC NAME]: [Child's name] is overdue for a vaccination. Vaccinating on time protects [Child's name] and other children who are too young to be vaccinated. To book or opt-out of SMS, call XXXX XXXX. Replies are not monitored, pls ignore SMS if you have an appt"

Figure S1: SMS examples. XX/XX/XX was populated by a date and call XXXX XXXX was populated by the clinic phone number.

2 Statistical Models

2.1 Day 28 vaccination

The primary model for SMS eligible index vaccinations was a Bayesian logistic regression model for 28-day vaccination status estimating the effect of each message type adjusting for vaccination age, clinic, and calendar time. Message effect and vaccination age parameters were given weakly informative priors on the log-odds scale. Site effects were assumed to be Normally distributed with shared variance. Calendar time effects were grouped into 4-week bins ("epochs") and given a random-walk prior of order 1. The full specification was,

$$
\begin{aligned}
Y_i &\sim \text{Bernoulli}(\pi_i) \\
\pi_i &= \text{logit}^{-1}(\eta_i) \\
\eta_i &= x_{\text{arm}_i}^{\mathsf{T}}\beta + w_{\text{age}_i}^{\mathsf{T}}\alpha + \gamma_{\text{clinic}_i} + \xi_{\text{epoch}_i} \\
\beta_1 &\sim \text{Normal}(\text{logit}^{-1}(0.8), 2.5^2) \\
\beta_2, \dots, \beta_{13} &\sim \text{Normal}(0, 1) \\
\alpha_1 \dots, \alpha_5 &\sim \text{Normal}(0, 2.5^2) \\
\gamma_c | \tau &\sim \text{Normal}(0, \tau^2), \quad c = 1, \dots, C \\
\xi_1 &= 0 \\
\xi_e | \omega &\sim \text{Normal}(\xi_{e-1}, \omega^2), \quad e = 2, \dots, E \\
\tau, \omega &\sim \text{Student-t}^+(3, 0, 1).
\end{aligned}
$$

To extend the model to all SMS eligible vaccinations for all children of participating parents, an additional hierarchical parent term was included in the linear predictor,

$$
\begin{aligned}
\eta_i &= x_{\text{arm}_i}^{\mathsf{T}}\beta + w_{\text{age}_i}^{\mathsf{T}}\alpha + \gamma_{\text{clinic}_i} + \xi_{\text{epoch}_i} + \zeta_{\text{parent}_i} \\
\zeta_p | \varrho &\sim \text{Normal}(0, \varrho^2), \quad p = 1, \dots, P \\
\varrho &\sim \text{Student-t}^+(3, 0, 1).
\end{aligned}
$$

For the primary model, the message design matrix, X, was saturated. Reduced design matrices were considered where: the interaction term between message timing and message framing was assumed to be zero, a model with only distinct timing effects which were assumed to apply to all content framings, a model with only distinct framing effects which were assumed to apply across all timings, and a completely pooled model where all message types shared the same effect.

To allow for heterogeneity of effect with respect to vaccination age, each of the above models were extended to allow for age-specific message effects, in which case

$$
\eta_i = x_{\text{arm}_i}^{\mathsf{T}}\beta_{\text{age}_i} + \gamma_{\text{clinic}_i} + \xi_{\text{epoch}_i}
$$

with all other model terms unchanged.

Each model was estimated by Markov chain Monte Carlo using 10,000 samples from the joint posterior.

2.2 Day of vaccination

To better understand differences in timing of vaccination by reminder type, secondary analyses used discrete-time logistic-hazard regression models to estimate the effect of each reminder type on the continuation ratio for day of vaccination receipt (from -14 to 28) relative to the scheduled due date (day 0), adjusting for source clinic, and calendar time (epoch) of the scheduled due date. The baseline hazard was stratified by age and smoothed using low rank thin plate regression splines with 8 basis functions. SMS reminder type was treated as a time-varying covariate, with all participants contributing to the no reminder hazard until the day on which their SMS was scheduled to be sent Reminder effects were assumed constant over time. Time-varying effects were considered plausible and exploratory analyses allowing for age-specific time-varying effects of reminder timings to be investigated. Differences in cumulative indicidence of vaccination to day 28 were estimated for the most recent epoch assuming a "typical" source clinic (and parent) where the random effect terms were equal to 0. Due to the spike in the hazard of vaccination on day 0, sensitivity analyses removed the smoothness assumption allowing for unstructured hazards by day but found little difference in estimates of message effects and are not reported here.

The full specification of the primary analysis for day of vaccination receipt for SMS eligible index vaccinations was,

$$
\begin{aligned}
\lambda_i(d) &= \Pr(D_i = d | D_i \geq d) \\
&= \text{logit}^{-1}(\eta_{id}) \\
\eta_{id} &= s_{\text{age}_i}(d) + x_{\text{arm}_i}(d)^\mathsf{T}\beta + \gamma_{\text{clinic}_i} + \xi_{\text{epoch}_i} \\
\beta_1, \dots, \beta_{12} &\sim \text{Normal}(0, 1) \\
s_{\text{age}_i}(d) &= \alpha_{\text{age},0} + \alpha_{\text{age},1} + \textstyle\sum_{m=2}^{M} \alpha_{\text{age},m} Z_{md} \\
\alpha_{\text{age},0}, \alpha_{\text{age},1} &\sim \text{Normal}(0, 2.5^2) \\
\alpha_{\text{age},m} | \varrho &\sim \text{Normal}(0, \varrho^2) \\
\gamma_c | \tau &\sim \text{Normal}(0, \tau^2) \\
\xi_1 &= 0 \\
\xi_e | \omega &\sim \text{Normal}(\xi_{e-1}, \omega^2) \\
\varrho, \tau, \omega &\sim \text{Student-t}^+(3, 0, 1)
\end{aligned}
$$

where $X(d)$ was the saturated time-varying treatment design, and Z was the penalised low-rank thin-plate spline design matrix. Similar to the analysis of day 28 vaccinations, reduced models were considered.

To allow for heterogeneity of effect with respesct to vaccination age, the model was extended to allow for age-specific message effects, in which case

$$
\eta_{id} = s_{\text{age}_i}(d) + x_{\text{arm}_i}(d)^\mathsf{T}\beta_{\text{age}_i} + \gamma_{\text{clinic}_i} + \xi_{\text{epoch}_i}.
$$

To allow for age-specific time-varying message effects, the model was further extended as

$$
\begin{aligned}
\eta_{id} &= s_{\text{age}_i}(d) + x_{\text{arm}_i}(d) f_{\text{age}_i,\text{arm}_i}(d) + \gamma_{\text{clinic}_i} + \xi_{\text{epoch}_i} \\
f_{\text{age}_i,\text{arm}_i}(d) &= \phi_{\text{age},\text{arm},0} + \phi_{\text{age},\text{arm},1} + \textstyle\sum_{m=2}^{M} \phi_{\text{age},\text{arm},m} Z_{md}
\end{aligned}
$$

Each model was estimated by Markov chain Monte Carlo using 4,000 samples from the joint posterior.

3 Baseline and Descriptive Statistics

Table S1: Summary of participating clinics.

Clinic	Clinic location	2021 SEIFA quintile[1]
1	VIC, Rural	2
2	TAS, Urban	1
3	NSW, Urban	4
4	ACT, Urban	5
5	NSW, Urban	3
6	NSW, Urban	3
7	QLD, Urban	5
8	WA, Urban	5
9	WA, Urban	5
10	VIC, Urban	5
11	NSW, Urban	3
12	NSW, Urban	4
13	QLD, Rural	3
14	WA, Urban	3
15	VIC, Urban	2
16	TAS, Rural	1
17	NSW, Urban	4
18	QLD, Rural	3
19	NT, Urban	5
20	VIC, Urban	5

[1]2021 Socio-economic index for areas (SEIFA), Index of Relative Socio-economic Disadvantage (IRSD) quintile, 1 = most disadvantaged, 5 = most advantaged

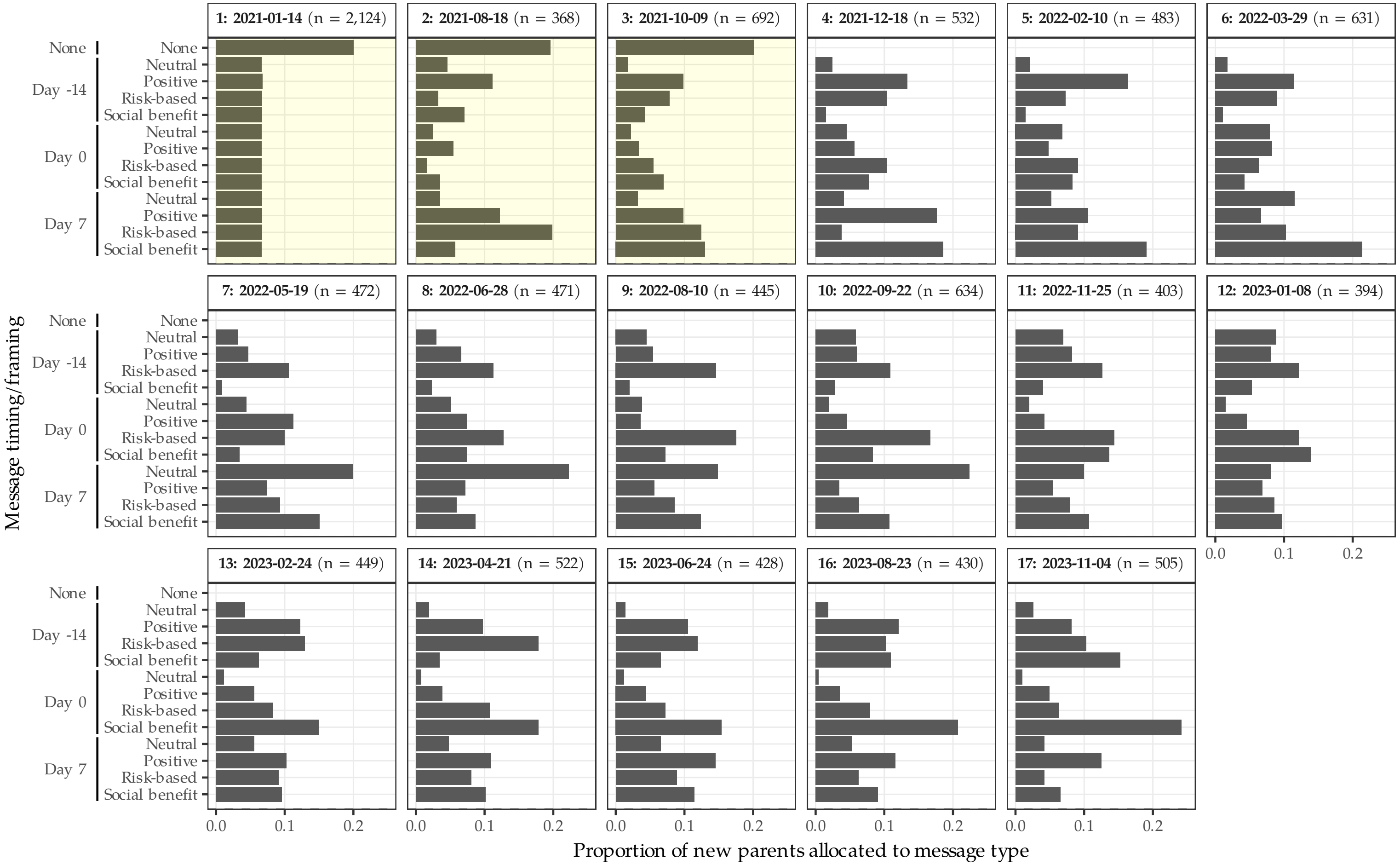

Figure S2: Proportion of newly encountered parents allocated to each message type by allocation cohort. The start date for each set of allocation proportions is given along with the total number of parents randomised. Vertical lines indicate target proportions under equal allocation without control group (1/12). Highlighted panels indicate control arm availability.

Table S2: Distribution of vaccination ages, participating clinic, and parent allocation cohort, by assigned message type, for SMS eligible index vaccinations.

		Day -14				Day 0				Day 7			
	None	Neutral	Positive	Risk-based	Social benefit	Neutral	Positive	Risk-based	Social benefit	Neutral	Positive	Risk-based	Social benefit
Assigned													
	637	409	893	985	483	380	560	905	984	898	878	811	1110
Age (months)													
2	70 (.11)	64 (.16)	143 (.16)	186 (.19)	85 (.18)	55 (.14)	100 (.18)	170 (.19)	190 (.19)	163 (.18)	143 (.16)	120 (.15)	194 (.17)
4	120 (.19)	74 (.18)	174 (.19)	159 (.16)	88 (.18)	74 (.19)	89 (.16)	167 (.18)	174 (.18)	153 (.17)	161 (.18)	150 (.18)	188 (.17)
6	55 (.09)	26 (.06)	45 (.05)	38 (.04)	25 (.05)	18 (.05)	34 (.06)	32 (.04)	55 (.06)	44 (.05)	46 (.05)	39 (.05)	54 (.05)
12	94 (.15)	45 (.11)	88 (.10)	101 (.10)	44 (.09)	39 (.10)	64 (.11)	75 (.08)	89 (.09)	98 (.11)	96 (.11)	69 (.09)	105 (.09)
18	109 (.17)	48 (.12)	102 (.11)	101 (.10)	62 (.13)	60 (.16)	59 (.11)	106 (.12)	97 (.10)	108 (.12)	90 (.10)	102 (.13)	136 (.12)
48	189 (.30)	152 (.37)	341 (.38)	400 (.41)	179 (.37)	134 (.35)	214 (.38)	355 (.39)	379 (.39)	332 (.37)	342 (.39)	331 (.41)	433 (.39)
Clinic													
1	0 (.00)	6 (.01)	26 (.03)	30 (.03)	10 (.02)	5 (.01)	8 (.01)	17 (.02)	30 (.03)	23 (.03)	13 (.01)	17 (.02)	21 (.02)
2	9 (.01)	5 (.01)	36 (.04)	32 (.03)	10 (.02)	4 (.01)	11 (.02)	17 (.02)	21 (.02)	23 (.03)	21 (.02)	29 (.04)	37 (.03)
3	49 (.08)	37 (.09)	61 (.07)	62 (.06)	35 (.07)	29 (.08)	41 (.07)	65 (.07)	63 (.06)	55 (.06)	50 (.06)	65 (.08)	78 (.07)
4	6 (.01)	5 (.01)	9 (.01)	1 (.00)	6 (.01)	0 (.00)	3 (.01)	8 (.01)	9 (.01)	3 (.00)	4 (.00)	6 (.01)	5 (.00)
5	23 (.04)	15 (.04)	76 (.09)	74 (.08)	25 (.05)	23 (.06)	35 (.06)	75 (.08)	79 (.08)	66 (.07)	65 (.07)	61 (.08)	116 (.10)
6	7 (.01)	6 (.01)	10 (.01)	7 (.01)	1 (.00)	3 (.01)	5 (.01)	5 (.01)	7 (.01)	7 (.01)	9 (.01)	8 (.01)	8 (.01)
7	7 (.01)	11 (.03)	24 (.03)	30 (.03)	13 (.03)	11 (.03)	18 (.03)	31 (.03)	22 (.02)	20 (.02)	22 (.03)	19 (.02)	41 (.04)
8	113 (.18)	50 (.12)	78 (.09)	93 (.09)	62 (.13)	56 (.15)	75 (.13)	89 (.10)	90 (.09)	98 (.11)	100 (.11)	106 (.13)	117 (.11)
9	53 (.08)	19 (.05)	52 (.06)	55 (.06)	21 (.04)	16 (.04)	33 (.06)	42 (.05)	49 (.05)	41 (.05)	51 (.06)	52 (.06)	58 (.05)
10	0 (.00)	18 (.04)	50 (.06)	79 (.08)	22 (.05)	21 (.06)	39 (.07)	62 (.07)	53 (.05)	76 (.08)	39 (.04)	60 (.07)	90 (.08)
11	92 (.14)	42 (.10)	97 (.11)	94 (.10)	58 (.12)	39 (.10)	49 (.09)	92 (.10)	96 (.10)	84 (.09)	91 (.10)	72 (.09)	107 (.10)
12	117 (.18)	72 (.18)	104 (.12)	130 (.13)	74 (.15)	61 (.16)	79 (.14)	103 (.11)	117 (.12)	110 (.12)	137 (.16)	97 (.12)	116 (.10)
13	4 (.01)	4 (.01)	14 (.02)	20 (.02)	0 (.00)	2 (.01)	5 (.01)	14 (.02)	23 (.02)	20 (.02)	13 (.01)	11 (.01)	17 (.02)
14	20 (.03)	6 (.01)	23 (.03)	24 (.02)	14 (.03)	11 (.03)	13 (.02)	17 (.02)	28 (.03)	26 (.03)	16 (.02)	14 (.02)	28 (.03)
15	0 (.00)	5 (.01)	23 (.03)	18 (.02)	18 (.04)	6 (.02)	9 (.02)	30 (.03)	33 (.03)	18 (.02)	25 (.03)	13 (.02)	14 (.01)
16	4 (.01)	7 (.02)	16 (.02)	12 (.01)	6 (.01)	2 (.01)	8 (.01)	17 (.02)	12 (.01)	8 (.01)	15 (.02)	12 (.01)	17 (.02)
17	127 (.20)	66 (.16)	109 (.12)	107 (.11)	67 (.14)	65 (.17)	74 (.13)	122 (.13)	111 (.11)	108 (.12)	114 (.13)	96 (.12)	118 (.11)
18	6 (.01)	4 (.01)	16 (.02)	22 (.02)	7 (.01)	4 (.01)	7 (.01)	17 (.02)	24 (.02)	23 (.03)	21 (.02)	18 (.02)	26 (.02)
19	0 (.00)	4 (.01)	16 (.02)	15 (.02)	5 (.01)	10 (.03)	12 (.02)	18 (.02)	26 (.03)	23 (.03)	15 (.02)	8 (.01)	19 (.02)
20	0 (.00)	27 (.07)	53 (.06)	80 (.08)	29 (.06)	12 (.03)	36 (.06)	64 (.07)	91 (.09)	66 (.07)	57 (.06)	47 (.06)	77 (.07)
Parent Cohort													
01 Jan 2021 - 17 Aug 2021	425 (.67)	142 (.35)	143 (.16)	142 (.14)	143 (.30)	141 (.37)	141 (.25)	142 (.16)	141 (.14)	142 (.16)	142 (.16)	142 (.18)	141 (.13)
18 Aug 2021 - 08 Oct 2021	73 (.11)	17 (.04)	42 (.05)	12 (.01)	26 (.05)	9 (.02)	20 (.04)	6 (.01)	13 (.01)	13 (.01)	45 (.05)	73 (.09)	22 (.02)
09 Oct 2021 - 17 Dec 2021	139 (.22)	12 (.03)	68 (.08)	54 (.05)	29 (.06)	15 (.04)	23 (.04)	38 (.04)	49 (.05)	22 (.02)	68 (.08)	86 (.11)	90 (.08)
18 Dec 2021 - 09 Feb 2022	0 (.00)	13 (.03)	71 (.08)	55 (.06)	8 (.02)	24 (.06)	30 (.05)	55 (.06)	41 (.04)	22 (.02)	94 (.11)	20 (.02)	99 (.09)
10 Feb 2022 - 28 Mar 2022	0 (.00)	10 (.02)	79 (.09)	35 (.04)	7 (.01)	33 (.09)	23 (.04)	43 (.05)	39 (.04)	25 (.03)	51 (.06)	44 (.05)	92 (.08)
29 Mar 2022 - 18 May 2022	0 (.00)	11 (.03)	72 (.08)	57 (.06)	7 (.01)	50 (.13)	52 (.09)	40 (.04)	27 (.03)	73 (.08)	42 (.05)	65 (.08)	135 (.12)
19 May 2022 - 27 Jun 2022	0 (.00)	15 (.04)	22 (.02)	50 (.05)	4 (.01)	21 (.06)	54 (.10)	47 (.05)	17 (.02)	94 (.10)	35 (.04)	44 (.05)	71 (.06)
28 Jun 2022 - 09 Aug 2022	0 (.00)	14 (.03)	31 (.03)	53 (.05)	10 (.02)	24 (.06)	35 (.06)	60 (.07)	35 (.04)	105 (.12)	34 (.04)	28 (.03)	41 (.04)
10 Aug 2022 - 21 Sep 2022	0 (.00)	20 (.05)	24 (.03)	65 (.07)	9 (.02)	17 (.04)	16 (.03)	78 (.09)	32 (.03)	67 (.07)	25 (.03)	38 (.05)	55 (.05)
22 Sep 2022 - 24 Nov 2022	0 (.00)	37 (.09)	38 (.04)	69 (.07)	18 (.04)	12 (.03)	29 (.05)	106 (.12)	53 (.05)	141 (.16)	22 (.03)	40 (.05)	68 (.06)
25 Nov 2022 - 07 Jan 2023	0 (.00)	28 (.07)	33 (.04)	51 (.05)	16 (.03)	8 (.02)	17 (.03)	58 (.06)	55 (.06)	40 (.04)	22 (.03)	32 (.04)	43 (.04)
08 Jan 2023 - 23 Feb 2023	0 (.00)	34 (.08)	32 (.04)	48 (.05)	21 (.04)	6 (.02)	18 (.03)	48 (.05)	55 (.06)	32 (.04)	27 (.03)	34 (.04)	38 (.03)
24 Feb 2023 - 20 Apr 2023	0 (.00)	19 (.05)	55 (.06)	58 (.06)	28 (.06)	5 (.01)	25 (.04)	37 (.04)	67 (.07)	25 (.03)	46 (.05)	40 (.05)	43 (.04)
21 Apr 2023 - 23 Jun 2023	0 (.00)	10 (.02)	51 (.06)	93 (.09)	18 (.04)	4 (.01)	20 (.04)	55 (.06)	93 (.09)	25 (.03)	56 (.06)	42 (.05)	53 (.05)
24 Jun 2023 - 22 Aug 2023	0 (.00)	6 (.01)	45 (.05)	51 (.05)	28 (.06)	5 (.01)	19 (.03)	31 (.03)	66 (.07)	28 (.03)	62 (.07)	38 (.05)	49 (.04)
23 Aug 2023 - 03 Nov 2023	0 (.00)	8 (.02)	51 (.06)	44 (.04)	46 (.10)	2 (.01)	15 (.03)	34 (.04)	89 (.09)	23 (.03)	50 (.06)	27 (.03)	39 (.04)
04 Nov 2023 - 25 Feb 2024	0 (.00)	13 (.03)	36 (.04)	48 (.05)	65 (.13)	4 (.01)	23 (.04)	27 (.03)	112 (.11)	21 (.02)	57 (.06)	18 (.02)	31 (.03)

Table S3: Distribution of vaccination ages, participating clinic, and parent allocation cohort, by assigned message type, for all SMS eligible vaccinations.

		Day -14				Day 0				Day 7			
	None	Neutral	Positive	Risk-based	Social benefit	Neutral	Positive	Risk-based	Social benefit	Neutral	Positive	Risk-based	Social benefit
Assigned													
	1766	950	1922	2009	953	895	1266	1915	1929	1943	1827	1763	2361
Age (months)													
2	98 (.06)	72 (.08)	167 (.09)	209 (.10)	91 (.10)	70 (.08)	118 (.09)	190 (.10)	214 (.11)	183 (.09)	157 (.09)	139 (.08)	223 (.09)
4	207 (.12)	121 (.13)	264 (.14)	267 (.13)	138 (.14)	112 (.13)	167 (.13)	274 (.14)	277 (.14)	259 (.13)	245 (.13)	241 (.14)	312 (.13)
6	268 (.15)	144 (.15)	306 (.16)	298 (.15)	143 (.15)	131 (.15)	198 (.16)	297 (.16)	322 (.17)	303 (.16)	284 (.16)	275 (.16)	365 (.15)
12	357 (.20)	180 (.19)	347 (.18)	350 (.17)	157 (.16)	168 (.19)	236 (.19)	328 (.17)	317 (.16)	367 (.19)	323 (.18)	307 (.17)	423 (.18)
18	461 (.26)	204 (.21)	396 (.21)	376 (.19)	184 (.19)	217 (.24)	262 (.21)	367 (.19)	331 (.17)	407 (.21)	368 (.20)	362 (.21)	486 (.21)
48	375 (.21)	229 (.24)	442 (.23)	509 (.25)	240 (.25)	197 (.22)	285 (.23)	459 (.24)	468 (.24)	424 (.22)	450 (.25)	439 (.25)	552 (.23)
Clinic													
1	0 (.00)	11 (.01)	59 (.03)	57 (.03)	14 (.01)	7 (.01)	14 (.01)	22 (.01)	46 (.02)	50 (.03)	18 (.01)	29 (.02)	35 (.01)
2	28 (.02)	17 (.02)	72 (.04)	68 (.03)	17 (.02)	8 (.01)	32 (.03)	35 (.02)	58 (.03)	61 (.03)	45 (.02)	63 (.04)	84 (.04)
3	138 (.08)	76 (.08)	133 (.07)	117 (.06)	76 (.08)	70 (.08)	95 (.08)	150 (.08)	134 (.07)	107 (.06)	101 (.06)	137 (.08)	170 (.07)
4	10 (.01)	8 (.01)	11 (.01)	1 (.00)	11 (.01)	0 (.00)	6 (.00)	9 (.00)	18 (.01)	4 (.00)	9 (.00)	13 (.01)	9 (.00)
5	79 (.04)	28 (.03)	167 (.09)	147 (.07)	40 (.04)	48 (.05)	86 (.07)	170 (.09)	153 (.08)	151 (.08)	155 (.08)	132 (.07)	250 (.11)
6	18 (.01)	14 (.01)	15 (.01)	18 (.01)	1 (.00)	7 (.01)	11 (.01)	13 (.01)	12 (.01)	10 (.01)	26 (.01)	15 (.01)	13 (.01)
7	19 (.01)	18 (.02)	63 (.03)	49 (.02)	18 (.02)	21 (.02)	42 (.03)	66 (.03)	45 (.02)	36 (.02)	43 (.02)	38 (.02)	93 (.04)
8	302 (.17)	110 (.12)	162 (.08)	232 (.12)	141 (.15)	157 (.18)	181 (.14)	209 (.11)	161 (.08)	219 (.11)	200 (.11)	245 (.14)	273 (.12)
9	181 (.10)	50 (.05)	142 (.07)	131 (.07)	44 (.05)	46 (.05)	76 (.06)	113 (.06)	120 (.06)	97 (.05)	105 (.06)	154 (.09)	138 (.06)
10	0 (.00)	44 (.05)	104 (.05)	138 (.07)	38 (.04)	47 (.05)	70 (.06)	105 (.05)	87 (.05)	133 (.07)	67 (.04)	110 (.06)	170 (.07)
11	236 (.13)	93 (.10)	226 (.12)	207 (.10)	114 (.12)	86 (.10)	104 (.08)	204 (.11)	175 (.09)	188 (.10)	196 (.11)	131 (.07)	218 (.09)
12	323 (.18)	176 (.19)	235 (.12)	274 (.14)	155 (.16)	149 (.17)	185 (.15)	194 (.10)	274 (.14)	247 (.13)	316 (.17)	217 (.12)	259 (.11)
13	8 (.00)	11 (.01)	22 (.01)	46 (.02)	0 (.00)	6 (.01)	10 (.01)	30 (.02)	42 (.02)	37 (.02)	31 (.02)	27 (.02)	40 (.02)
14	49 (.03)	20 (.02)	52 (.03)	47 (.02)	26 (.03)	28 (.03)	34 (.03)	29 (.02)	63 (.03)	59 (.03)	37 (.02)	26 (.01)	50 (.02)
15	0 (.00)	6 (.01)	28 (.01)	23 (.01)	24 (.03)	6 (.01)	11 (.01)	37 (.02)	40 (.02)	20 (.01)	35 (.02)	18 (.01)	19 (.01)
16	6 (.00)	16 (.02)	26 (.01)	23 (.01)	16 (.02)	3 (.00)	20 (.02)	43 (.02)	15 (.01)	25 (.01)	22 (.01)	26 (.01)	49 (.02)
17	355 (.20)	180 (.19)	244 (.13)	214 (.11)	145 (.15)	151 (.17)	165 (.13)	282 (.15)	238 (.12)	261 (.13)	249 (.14)	229 (.13)	250 (.11)
18	14 (.01)	13 (.01)	37 (.02)	58 (.03)	17 (.02)	13 (.01)	25 (.02)	39 (.02)	63 (.03)	75 (.04)	61 (.03)	46 (.03)	62 (.03)
19	0 (.00)	4 (.00)	21 (.01)	22 (.01)	5 (.01)	13 (.01)	17 (.01)	25 (.01)	36 (.02)	37 (.02)	15 (.01)	16 (.01)	27 (.01)
20	0 (.00)	55 (.06)	103 (.05)	137 (.07)	51 (.05)	29 (.03)	82 (.06)	140 (.07)	149 (.08)	126 (.06)	96 (.05)	91 (.05)	152 (.06)
Parent Cohort													
01 Jan 2021 - 17 Aug 2021	1203 (.68)	421 (.44)	410 (.21)	436 (.22)	375 (.39)	385 (.43)	398 (.31)	407 (.21)	404 (.21)	417 (.21)	421 (.23)	405 (.23)	412 (.17)
18 Aug 2021 - 08 Oct 2021	171 (.10)	40 (.04)	117 (.06)	27 (.01)	56 (.06)	28 (.03)	58 (.05)	10 (.01)	32 (.02)	39 (.02)	125 (.07)	175 (.10)	48 (.02)
09 Oct 2021 - 17 Dec 2021	392 (.22)	28 (.03)	190 (.10)	122 (.06)	70 (.07)	42 (.05)	71 (.06)	81 (.04)	134 (.07)	49 (.03)	165 (.09)	215 (.12)	230 (.10)
18 Dec 2021 - 09 Feb 2022	0 (.00)	29 (.03)	152 (.08)	131 (.07)	10 (.01)	51 (.06)	70 (.06)	141 (.07)	90 (.05)	49 (.03)	211 (.12)	62 (.04)	242 (.10)
10 Feb 2022 - 28 Mar 2022	0 (.00)	22 (.02)	197 (.10)	73 (.04)	23 (.02)	80 (.09)	56 (.04)	117 (.06)	94 (.05)	71 (.04)	111 (.06)	90 (.05)	193 (.08)
29 Mar 2022 - 18 May 2022	0 (.00)	21 (.02)	162 (.08)	145 (.07)	15 (.02)	118 (.13)	114 (.09)	90 (.05)	68 (.04)	165 (.08)	87 (.05)	141 (.08)	278 (.12)
19 May 2022 - 27 Jun 2022	0 (.00)	43 (.05)	59 (.03)	105 (.05)	12 (.01)	50 (.06)	120 (.09)	86 (.04)	41 (.02)	190 (.10)	70 (.04)	109 (.06)	144 (.06)
28 Jun 2022 - 09 Aug 2022	0 (.00)	29 (.03)	60 (.03)	98 (.05)	16 (.02)	42 (.05)	74 (.06)	139 (.07)	76 (.04)	231 (.12)	79 (.04)	50 (.03)	80 (.03)
10 Aug 2022 - 21 Sep 2022	0 (.00)	41 (.04)	43 (.02)	120 (.06)	17 (.02)	29 (.03)	31 (.02)	162 (.08)	74 (.04)	139 (.07)	49 (.03)	81 (.05)	118 (.05)
22 Sep 2022 - 24 Nov 2022	0 (.00)	91 (.10)	80 (.04)	141 (.07)	42 (.04)	20 (.02)	61 (.05)	208 (.11)	116 (.06)	263 (.14)	44 (.02)	65 (.04)	133 (.06)
25 Nov 2022 - 07 Jan 2023	0 (.00)	54 (.06)	49 (.03)	90 (.04)	32 (.03)	11 (.01)	29 (.02)	110 (.06)	104 (.05)	79 (.04)	43 (.02)	63 (.04)	82 (.03)
08 Jan 2023 - 23 Feb 2023	0 (.00)	55 (.06)	53 (.03)	80 (.04)	36 (.04)	11 (.01)	36 (.03)	92 (.05)	103 (.05)	65 (.03)	49 (.03)	53 (.03)	75 (.03)
24 Feb 2023 - 20 Apr 2023	0 (.00)	33 (.03)	92 (.05)	99 (.05)	47 (.05)	9 (.01)	33 (.03)	56 (.03)	107 (.06)	45 (.02)	67 (.04)	71 (.04)	90 (.04)
21 Apr 2023 - 23 Jun 2023	0 (.00)	13 (.01)	74 (.04)	142 (.07)	27 (.03)	6 (.01)	34 (.03)	87 (.05)	134 (.07)	42 (.02)	85 (.05)	65 (.04)	83 (.04)
24 Jun 2023 - 22 Aug 2023	0 (.00)	6 (.01)	65 (.03)	78 (.04)	41 (.04)	7 (.01)	34 (.03)	45 (.02)	98 (.05)	44 (.02)	87 (.05)	61 (.03)	73 (.03)
23 Aug 2023 - 03 Nov 2023	0 (.00)	11 (.01)	79 (.04)	70 (.03)	63 (.07)	2 (.00)	22 (.02)	49 (.03)	130 (.07)	33 (.02)	70 (.04)	34 (.02)	45 (.02)
04 Nov 2023 - 25 Feb 2024	0 (.00)	13 (.01)	40 (.02)	52 (.03)	71 (.07)	4 (.00)	25 (.02)	35 (.02)	124 (.06)	22 (.01)	64 (.04)	23 (.01)	35 (.01)

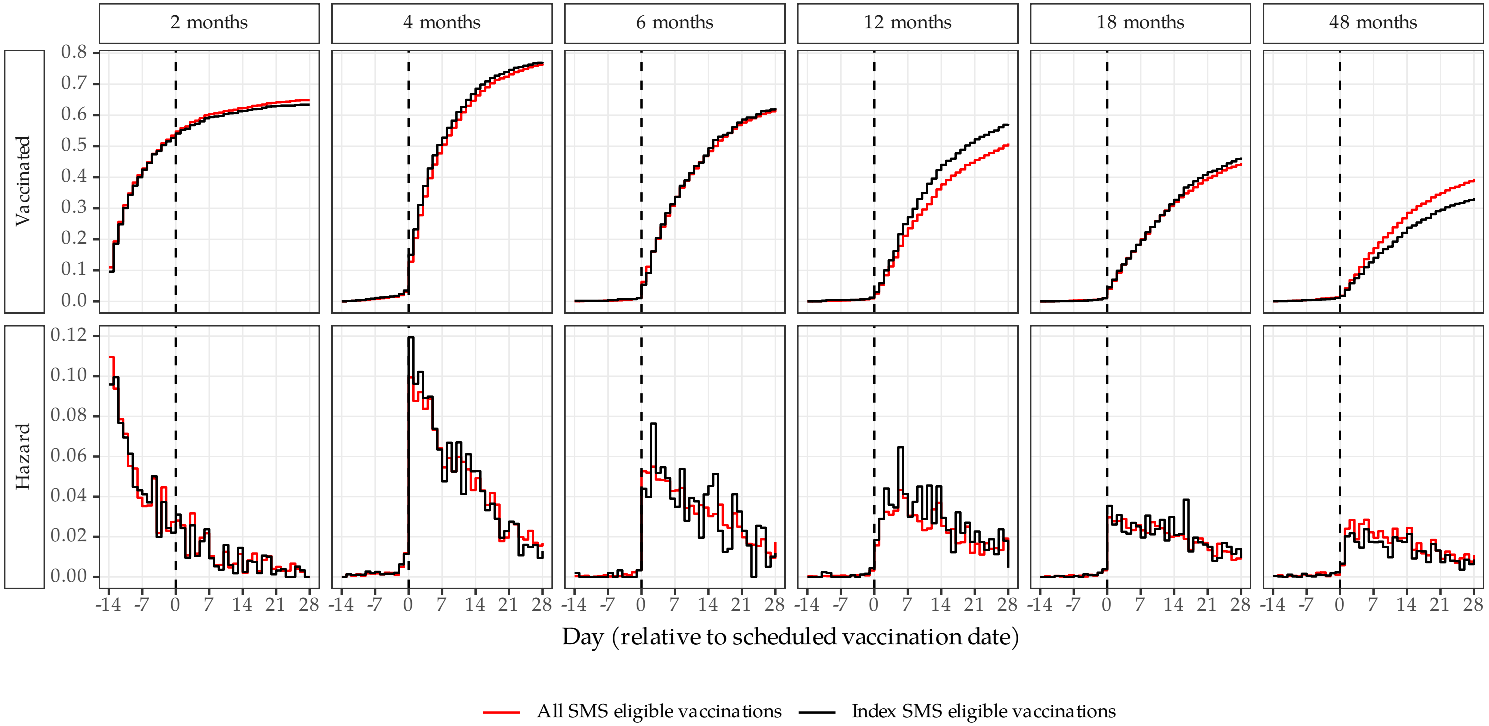

Figure S3: Cumulative proportion of vaccinations administered and empirical hazard by age for SMS eligible index vaccinations, and all SMS eligible vaccinations.

4 Statistical Analysis - Day 28 Vaccination Results

4.1 SMS eligible index vaccinations

Table S4: Posterior summary of message effect difference in proportion vaccinated by day 28 relative to no message amongst SMS eligible index vaccinations, by assumed model. Proportions are calculated with respect to a "typical" clinic (zero-effect), and a simple (unweighted) average over all vaccination ages.

	**Full (primary)**		**No interaction**		**Timing only**		**Framing only**		**Shared**	
	Median (95% CrI)	Pr(>0)	Median (95% CrI)	Pr(>0)	Median (95% CrI)	Pr(>0)	Median (95% CrI)	Pr(>0)	Median (95% CrI)	Pr(>0)
Any										
Any									0.06 (0.02, 0.11)	0.99
Day -14										
Neutral	0.01 (-0.05, 0.08)	0.64	0.03 (-0.03, 0.08)	0.85	0.06 (0.01, 0.10)	0.99	0.03 (-0.02, 0.09)	0.90		
Positive	0.08 (0.02, 0.13)	1.00	0.06 (0.01, 0.11)	0.99			0.07 (0.02, 0.12)	1.00		
Risk-based	0.08 (0.02, 0.13)	1.00	0.06 (0.01, 0.11)	0.99			0.06 (0.02, 0.11)	0.99		
Social benefit	0.04 (-0.03, 0.10)	0.88	0.06 (0.01, 0.11)	0.99			0.07 (0.02, 0.12)	1.00		
Day 0										
Neutral	0.00 (-0.06, 0.07)	0.55	0.04 (-0.02, 0.09)	0.92	0.07 (0.02, 0.12)	1.00				
Positive	0.06 (0.00, 0.12)	0.98	0.07 (0.02, 0.12)	1.00						
Risk-based	0.07 (0.01, 0.13)	0.99	0.07 (0.02, 0.12)	1.00						
Social benefit	0.10 (0.05, 0.16)	1.00	0.07 (0.02, 0.12)	1.00						
Day 7										
Neutral	0.07 (0.01, 0.12)	0.99	0.04 (-0.01, 0.09)	0.92	0.06 (0.01, 0.11)	0.99				
Positive	0.07 (0.02, 0.13)	1.00	0.07 (0.02, 0.12)	1.00						
Risk-based	0.06 (0.01, 0.12)	0.99	0.07 (0.02, 0.12)	0.99						
Social benefit	0.06 (0.01, 0.12)	0.99	0.07 (0.02, 0.12)	1.00						

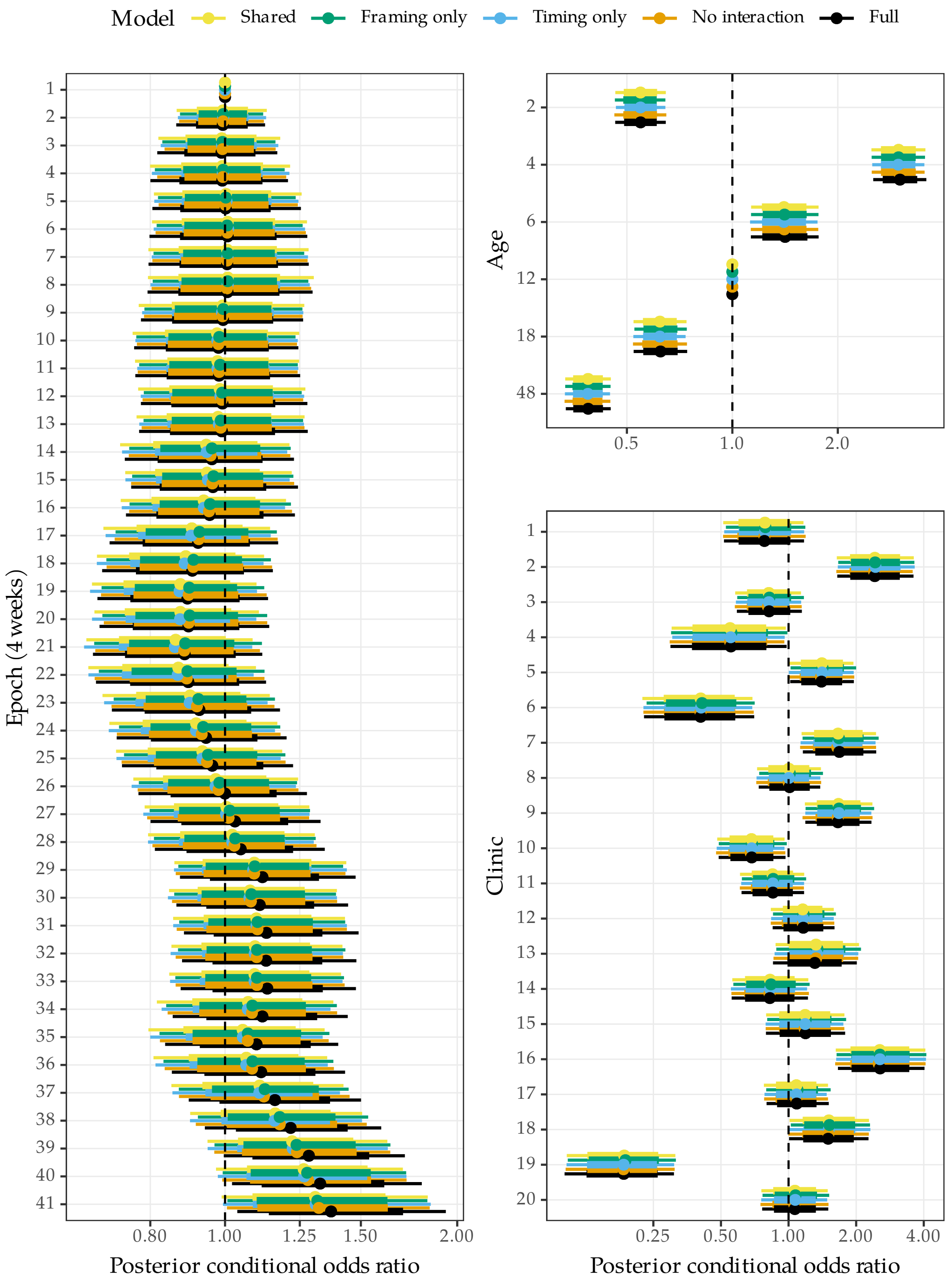

Figure S4: Posterior summaries of age, clinic, and epoch odds ratios for day 28 vaccination status amongst SMS eligible index vaccinations, by assumed model. Points – median, rectangles – 80% CrI, lines – 95% CrI.

4.2 All SMS eligible vaccinations

Table S5: Posterior summary of message effect odds ratios relative to no message for day 28 vaccination status amongst all SMS eligible vaccinations, by assumed model.

	**Full (primary)**		**No interaction**		**Timing only**		**Framing only**		**Shared**	
	Median (95% ETI)	Pr(>1)	Median (95% ETI)	Pr(>1)	Median (95% ETI)	Pr(>1)	Median (95% ETI)	Pr(>1)	Median (95% ETI)	Pr(>1)
Any										
Any									1.24 (1.03, 1.49)	0.99
Day -14										
Neutral	0.87 (0.65, 1.16)	0.17	1.06 (0.84, 1.33)	0.68	1.20 (0.98, 1.48)	0.96	1.10 (0.89, 1.36)	0.81		
Positive	1.42 (1.12, 1.82)	1.00	1.29 (1.04, 1.62)	0.99			1.34 (1.09, 1.65)	1.00		
Risk-based	1.27 (1.00, 1.60)	0.97	1.20 (0.96, 1.49)	0.95			1.24 (1.01, 1.52)	0.98		
Social benefit	1.08 (0.81, 1.44)	0.69	1.17 (0.93, 1.48)	0.91			1.22 (0.99, 1.51)	0.97		
Day 0										
Neutral	1.06 (0.78, 1.43)	0.65	1.11 (0.88, 1.41)	0.81	1.25 (1.01, 1.53)	0.98				
Positive	1.27 (0.96, 1.66)	0.96	1.36 (1.09, 1.70)	1.00						
Risk-based	1.28 (1.01, 1.63)	0.98	1.27 (1.02, 1.56)	0.98						
Social benefit	1.31 (1.03, 1.68)	0.99	1.23 (0.99, 1.53)	0.97						
Day 7										
Neutral	1.27 (0.99, 1.62)	0.97	1.12 (0.90, 1.41)	0.85	1.25 (1.02, 1.53)	0.98				
Positive	1.33 (1.04, 1.69)	0.99	1.37 (1.11, 1.72)	1.00						
Risk-based	1.21 (0.94, 1.53)	0.93	1.28 (1.03, 1.59)	0.99						
Social benefit	1.23 (0.99, 1.55)	0.96	1.24 (1.00, 1.55)	0.98						

Table S6: Posterior summary of message effect difference in proportion vaccinated by day 28 relative to no message amongst all SMS eligible vaccinations, by assumed model. Proportions are calculated with respect to a "typical" clinic and parent (zero-effect), and a simple (unweighted) average over all vaccination ages.

	**Full (primary)**		**No interaction**		**Timing only**		**Framing only**		**Shared**	
	Median (95% CrI)	Pr(>0)	Median (95% CrI)	Pr(>0)	Median (95% CrI)	Pr(>0)	Median (95% CrI)	Pr(>0)	Median (95% CrI)	Pr(>0)
Any										
Any									0.05 (0.01, 0.10)	0.99
Day -14										
Neutral	-0.03 (-0.10, 0.04)	0.17	0.01 (-0.04, 0.07)	0.68	0.04 (-0.01, 0.10)	0.96	0.02 (-0.03, 0.07)	0.81		
Positive	0.09 (0.03, 0.15)	1.00	0.06 (0.01, 0.12)	0.99			0.07 (0.02, 0.12)	1.00		
Risk-based	0.06 (0.00, 0.12)	0.97	0.05 (-0.01, 0.10)	0.95			0.05 (0.00, 0.10)	0.98		
Social benefit	0.02 (-0.05, 0.09)	0.69	0.04 (-0.02, 0.10)	0.91			0.05 (0.00, 0.10)	0.97		
Day 0										
Neutral	0.01 (-0.06, 0.09)	0.65	0.03 (-0.03, 0.08)	0.81	0.05 (0.00, 0.10)	0.98				
Positive	0.06 (-0.01, 0.12)	0.96	0.08 (0.02, 0.13)	1.00						
Risk-based	0.06 (0.00, 0.12)	0.98	0.06 (0.00, 0.11)	0.98						
Social benefit	0.07 (0.01, 0.13)	0.99	0.05 (0.00, 0.10)	0.97						
Day 7										
Neutral	0.06 (0.00, 0.12)	0.97	0.03 (-0.02, 0.08)	0.85	0.05 (0.00, 0.10)	0.98				
Positive	0.07 (0.01, 0.13)	0.99	0.08 (0.03, 0.13)	1.00						
Risk-based	0.05 (-0.02, 0.10)	0.93	0.06 (0.01, 0.11)	0.99						
Social benefit	0.05 (0.00, 0.11)	0.96	0.05 (0.00, 0.11)	0.98						

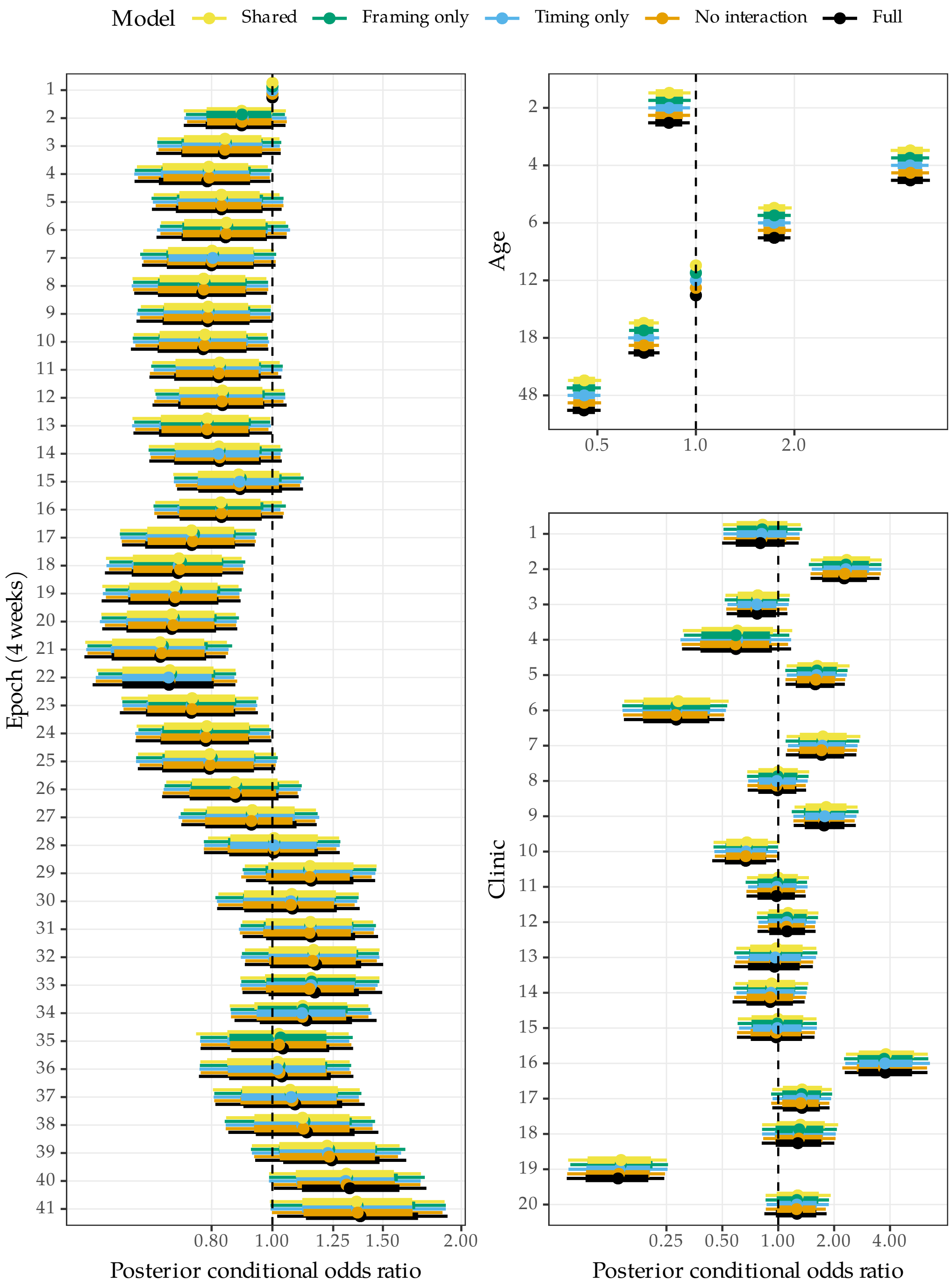

Figure S5: Posterior summaries of age, clinic, and epoch odds ratios for day 28 vaccination status amongst all SMS eligible vaccinations, by assumed model. Points – median, rectangles – 80% CrI, lines – 95% CrI.

4.3 SMS eligible index vaccinations amongst parents randomised contemporaneous with control

Table S7: Posterior summary of message effect odds ratios relative to no message for day 28 vaccination status amongst index SMS eligible vaccinations for parents randomised while control arm available, by assumed model.

	**Full (primary)**		**No interaction**		**Timing only**		**Framing only**		**Shared**	
	Median (95% ETI)	Pr(>1)	Median (95% ETI)	Pr(>1)	Median (95% ETI)	Pr(>1)	Median (95% ETI)	Pr(>1)	Median (95% ETI)	Pr(>1)
Any										
Any									1.33 (1.09, 1.60)	1.00
Day -14										
Neutral	1.04 (0.72, 1.49)	0.58	1.04 (0.78, 1.38)	0.61	1.21 (0.96, 1.51)	0.95	1.13 (0.89, 1.46)	0.83		
Positive	1.48 (1.07, 2.04)	0.99	1.24 (0.96, 1.62)	0.95			1.36 (1.08, 1.73)	1.00		
Risk-based	1.32 (0.94, 1.84)	0.95	1.29 (0.99, 1.70)	0.97			1.43 (1.13, 1.82)	1.00		
Social benefit	0.96 (0.68, 1.36)	0.41	1.22 (0.93, 1.60)	0.93			1.34 (1.06, 1.70)	0.99		
Day 0										
Neutral	1.15 (0.80, 1.65)	0.76	1.11 (0.84, 1.47)	0.76	1.28 (1.02, 1.61)	0.98				
Positive	1.18 (0.82, 1.69)	0.82	1.33 (1.01, 1.74)	0.98						
Risk-based	1.43 (1.00, 2.06)	0.98	1.38 (1.05, 1.82)	0.99						
Social benefit	1.37 (0.97, 1.93)	0.96	1.31 (0.99, 1.71)	0.97						
Day 7										
Neutral	1.23 (0.86, 1.76)	0.88	1.27 (0.96, 1.67)	0.95	1.47 (1.18, 1.84)	1.00				
Positive	1.40 (1.01, 1.94)	0.98	1.52 (1.16, 1.97)	1.00						
Risk-based	1.53 (1.13, 2.09)	1.00	1.57 (1.22, 2.05)	1.00						
Social benefit	1.73 (1.26, 2.39)	1.00	1.49 (1.14, 1.93)	1.00						

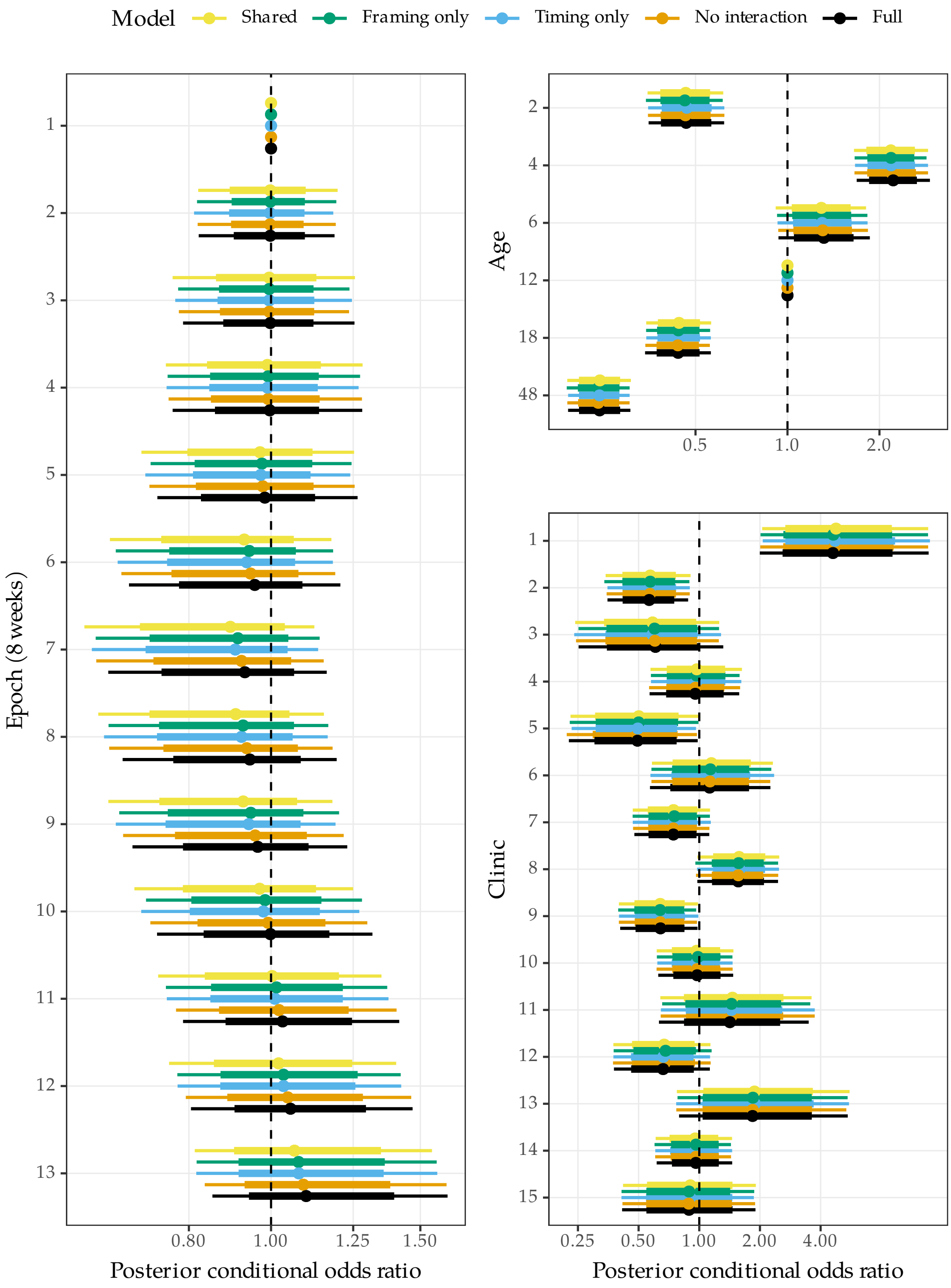

Figure S6: Posterior summaries of age, clinic, and epoch odds ratios for day 28 vaccination status amongst index SMS eligible vaccinations for parents randomised while control arm available, by assumed model. Points – median, rectangles – 80% CrI, lines – 95% CrI.

4.4 All SMS eligible vaccinations amongst parents randomised contemporaneous with control

Table S8: Posterior summary of message effect odds ratios relative to no message for day 28 vaccination status amongst all SMS eligible vaccinations for parents randomised while control arm available, by assumed model.

	**Full (primary)**		**No interaction**		**Timing only**		**Framing only**		**Shared**	
	Median (95% ETI)	Pr(>1)	Median (95% ETI)	Pr(>1)	Median (95% ETI)	Pr(>1)	Median (95% ETI)	Pr(>1)	Median (95% ETI)	Pr(>1)
Any										
Any									1.32 (1.07, 1.62)	0.99
Day -14										
Neutral	1.04 (0.69, 1.55)	0.57	1.11 (0.81, 1.54)	0.75	1.23 (0.96, 1.59)	0.95	1.20 (0.90, 1.59)	0.90		
Positive	1.57 (1.10, 2.28)	1.00	1.37 (1.02, 1.82)	0.98			1.46 (1.12, 1.88)	1.00		
Risk-based	1.34 (0.91, 2.00)	0.93	1.18 (0.87, 1.59)	0.86			1.27 (0.98, 1.66)	0.97		
Social benefit	0.94 (0.64, 1.38)	0.39	1.25 (0.91, 1.71)	0.92			1.35 (1.02, 1.77)	0.98		
Day 0										
Neutral	1.30 (0.86, 1.96)	0.89	1.23 (0.90, 1.70)	0.90	1.35 (1.03, 1.75)	0.99				
Positive	1.42 (0.96, 2.11)	0.96	1.50 (1.11, 2.04)	0.99						
Risk-based	1.25 (0.85, 1.84)	0.86	1.30 (0.96, 1.76)	0.95						
Social benefit	1.41 (0.96, 2.06)	0.96	1.38 (1.02, 1.88)	0.98						
Day 7										
Neutral	1.25 (0.84, 1.84)	0.86	1.25 (0.92, 1.71)	0.93	1.38 (1.07, 1.75)	0.99				
Positive	1.37 (0.95, 1.96)	0.95	1.52 (1.14, 2.04)	1.00						
Risk-based	1.25 (0.89, 1.72)	0.89	1.32 (0.99, 1.76)	0.97						
Social benefit	1.69 (1.19, 2.43)	1.00	1.40 (1.04, 1.90)	0.99						

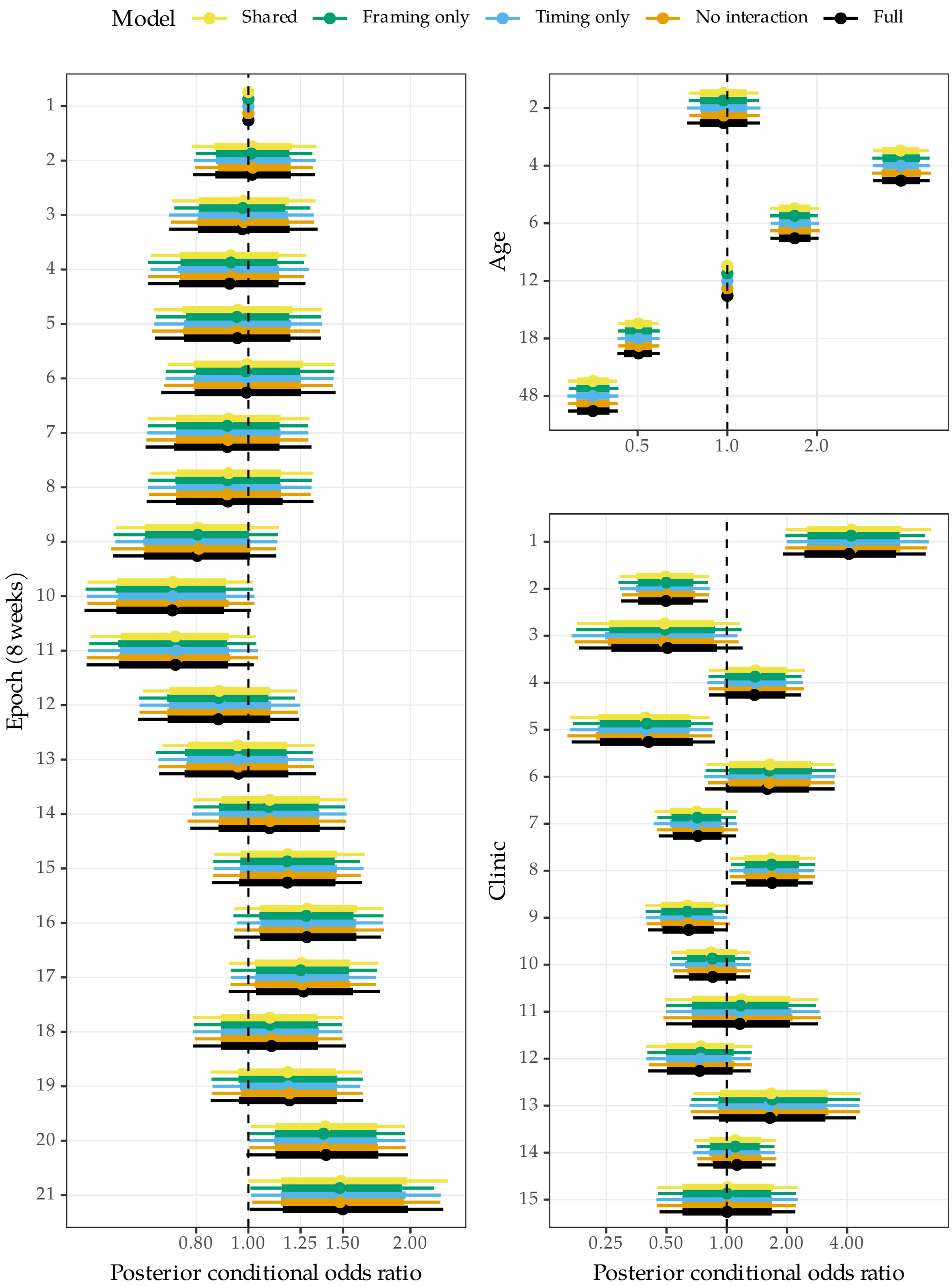

Figure S7: Posterior summaries of age, clinic, and epoch odds ratios for day 28 vaccination status amongst all SMS eligible vaccinations for parents randomised while control arm available, by assumed model. Points – median, rectangles – 80% CrI, lines – 95% CrI.

4.5 Age-specific effects

Table S9: Posterior summary (median and 95% credible interval) of age-specific odds ratio and difference in proportion vaccinated by day 28 (for typical clinic) of message relative to no message under shared effect model, for index vaccinations and all SMS eligible vaccinations.

	**Index vaccinations**			**All SMS eligible vaccinations**		
Age	Odds ratio	Pr(>1)	Risk difference	Odds ratio	Pr(>1)	Risk difference
2-month	1.12 (0.69, 1.82)	0.67	0.03 (-0.09, 0.13)	1.10 (0.66, 1.86)	0.63	0.02 (-0.10, 0.14)
4-month	1.08 (0.68, 1.62)	0.62	0.01 (-0.06, 0.10)	0.94 (0.61, 1.47)	0.39	-0.01 (-0.07, 0.07)
6-month	1.10 (0.63, 1.92)	0.64	0.02 (-0.10, 0.16)	1.00 (0.70, 1.44)	0.50	0.00 (-0.08, 0.09)
12-month	0.85 (0.55, 1.31)	0.23	-0.04 (-0.15, 0.07)	0.91 (0.66, 1.23)	0.27	-0.02 (-0.10, 0.05)
18-month	1.57 (1.04, 2.40)	0.98	0.10 (0.01, 0.19)	1.56 (1.18, 2.09)	1.00	0.10 (0.04, 0.16)
48-month	1.74 (1.21, 2.51)	1.00	0.10 (0.04, 0.16)	1.46 (1.06, 2.00)	0.99	0.07 (0.01, 0.12)
**Simple average**			0.04 (-0.01, 0.08)			0.03 (-0.02, 0.07)

Table S10: Posterior summary (median and 95% credible interval) of age-specific odds ratio and difference in proportion vaccinated by day 28 (for typical clinic) of message type relative to no message under timing-only effect model, for index vaccinations and all SMS eligible vaccinations.

	**Index vaccinations**			**All SMS eligible vaccinations**		
**Timing**	Odds ratio	Pr(>1)	Risk difference	Odds ratio	Pr(>1)	Risk difference
2-month						
Day -14	1.11 (0.66, 1.89)	0.66	0.02 (-0.10, 0.14)	1.08 (0.61, 1.93)	0.61	0.02 (-0.12, 0.15)
Day 0	1.13 (0.67, 1.90)	0.68	0.03 (-0.10, 0.14)	1.05 (0.60, 1.85)	0.57	0.01 (-0.12, 0.14)
Day 7	1.15 (0.69, 1.91)	0.70	0.03 (-0.09, 0.14)	1.17 (0.67, 2.04)	0.70	0.04 (-0.10, 0.16)
4-month						
Day -14	1.12 (0.68, 1.78)	0.68	0.02 (-0.06, 0.11)	0.95 (0.59, 1.51)	0.41	-0.01 (-0.08, 0.07)
Day 0	1.01 (0.62, 1.63)	0.52	0.00 (-0.08, 0.10)	0.98 (0.62, 1.57)	0.47	0.00 (-0.07, 0.08)
Day 7	1.09 (0.67, 1.74)	0.65	0.02 (-0.07, 0.11)	0.91 (0.56, 1.44)	0.34	-0.02 (-0.09, 0.06)
6-month						
Day -14	0.93 (0.49, 1.74)	0.41	-0.02 (-0.17, 0.14)	0.93 (0.63, 1.37)	0.35	-0.02 (-0.11, 0.08)
Day 0	1.23 (0.66, 2.31)	0.75	0.05 (-0.10, 0.20)	1.00 (0.67, 1.45)	0.50	0.00 (-0.09, 0.09)
Day 7	1.13 (0.61, 2.07)	0.66	0.03 (-0.11, 0.17)	1.07 (0.73, 1.57)	0.63	0.02 (-0.07, 0.11)
12-month						
Day -14	0.90 (0.56, 1.45)	0.32	-0.03 (-0.14, 0.09)	0.91 (0.64, 1.29)	0.30	-0.02 (-0.11, 0.06)
Day 0	0.93 (0.58, 1.49)	0.38	-0.02 (-0.13, 0.10)	0.87 (0.62, 1.21)	0.21	-0.03 (-0.12, 0.05)
Day 7	0.76 (0.48, 1.21)	0.12	-0.07 (-0.18, 0.05)	0.93 (0.67, 1.31)	0.35	-0.02 (-0.10, 0.07)
18-month						
Day -14	1.40 (0.90, 2.23)	0.93	0.07 (-0.02, 0.17)	1.52 (1.12, 2.07)	1.00	0.09 (0.02, 0.16)
Day 0	1.73 (1.11, 2.72)	0.99	0.13 (0.02, 0.22)	1.59 (1.18, 2.18)	1.00	0.10 (0.04, 0.17)
Day 7	1.56 (1.03, 2.44)	0.98	0.10 (0.01, 0.19)	1.59 (1.18, 2.16)	1.00	0.10 (0.04, 0.16)
48-month						
Day -14	1.70 (1.20, 2.51)	1.00	0.10 (0.04, 0.16)	1.42 (1.02, 1.99)	0.98	0.06 (0.00, 0.12)
Day 0	1.76 (1.23, 2.56)	1.00	0.10 (0.04, 0.16)	1.53 (1.09, 2.16)	0.99	0.08 (0.02, 0.14)
Day 7	1.77 (1.24, 2.60)	1.00	0.10 (0.04, 0.17)	1.43 (1.03, 2.00)	0.98	0.06 (0.01, 0.12)
Simple average						
Day -14			0.03 (-0.02, 0.08)			0.02 (-0.03, 0.07)
Day 0			0.05 (0.00, 0.10)			0.03 (-0.02, 0.07)
Day 7			0.04 (-0.01, 0.08)			0.03 (-0.01, 0.08)

5 Statistical Analysis - Day of Vaccination Results

Table S11: Estimated message continuation ratios relative to no message for day of vaccination amongst SMS eligible index vaccinations.

	**Full (primary)**		**No interaction**		**Timing only**		**Shared**	
	Median (95% ETI)	Pr(>1)	Median (95% ETI)	Pr(>1)	Median (95% ETI)	Pr(>1)	Median (95% ETI)	Pr(>1)
Any								
Any							1.29 (1.20, 1.39)	1.00
Day -14								
Neutral	1.04 (0.87, 1.23)	0.66	1.11 (0.99, 1.25)	0.96	1.23 (1.14, 1.35)	1.00		
Positive	1.32 (1.17, 1.48)	1.00	1.28 (1.15, 1.41)	1.00				
Risk-based	1.29 (1.15, 1.44)	1.00	1.25 (1.13, 1.37)	1.00				
Social benefit	1.16 (1.00, 1.35)	0.97	1.24 (1.11, 1.39)	1.00				
Day 0								
Neutral	1.11 (0.92, 1.32)	0.86	1.21 (1.07, 1.38)	1.00	1.35 (1.22, 1.47)	1.00		
Positive	1.31 (1.11, 1.53)	1.00	1.39 (1.24, 1.55)	1.00				
Risk-based	1.34 (1.18, 1.53)	1.00	1.36 (1.22, 1.51)	1.00				
Social benefit	1.47 (1.30, 1.67)	1.00	1.36 (1.22, 1.51)	1.00				
Day 7								
Neutral	1.52 (1.28, 1.79)	1.00	1.33 (1.15, 1.53)	1.00	1.46 (1.31, 1.64)	1.00		
Positive	1.54 (1.30, 1.82)	1.00	1.53 (1.34, 1.73)	1.00				
Risk-based	1.41 (1.18, 1.66)	1.00	1.49 (1.31, 1.70)	1.00				
Social benefit	1.39 (1.19, 1.63)	1.00	1.49 (1.30, 1.69)	1.00				

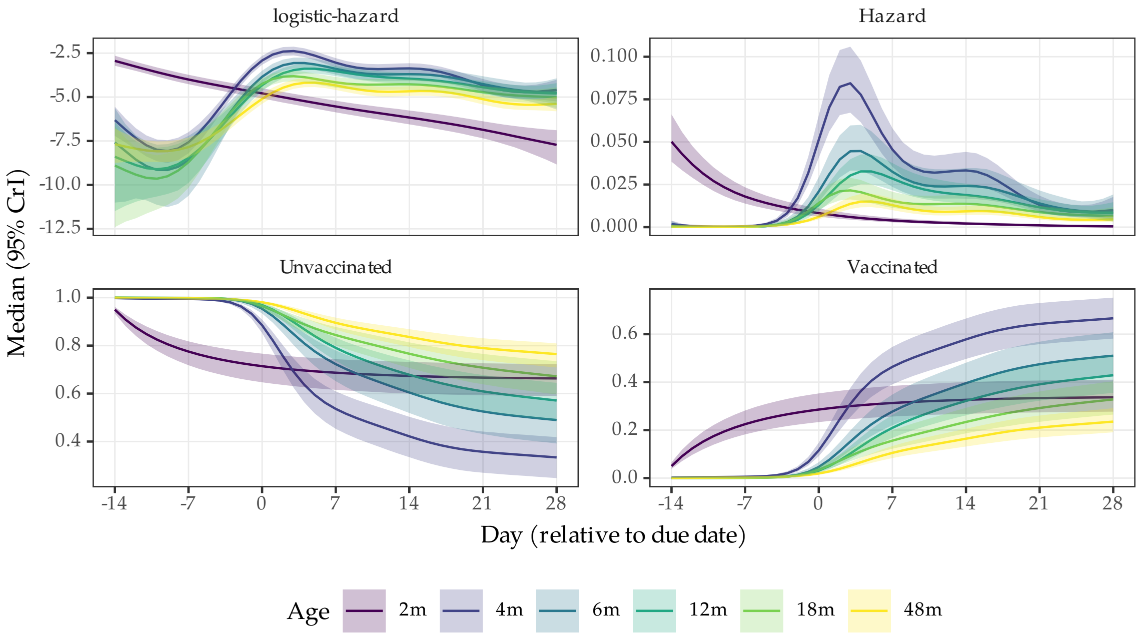

Figure S8: Estimated baseline hazard (no message) under the full model by scheduled vaccination age, typical clinic.

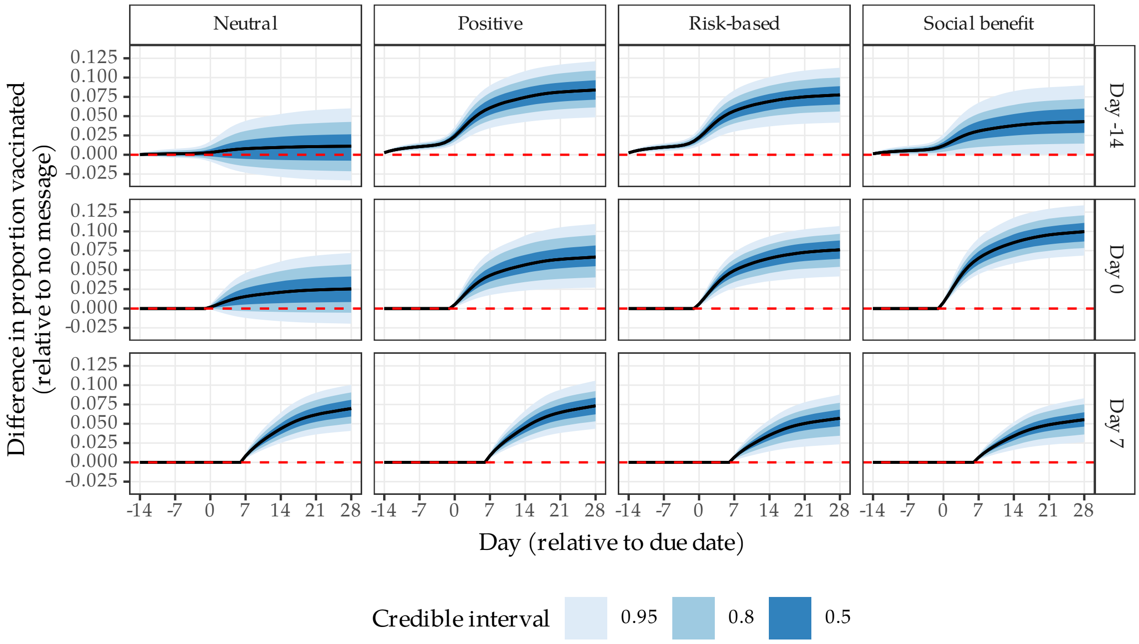

Figure S9: Posterior difference in probability of vaccination by day relative to scheduled due date for a "typical" clinic (zero-effect) and simple average across all vaccination ages under the full model with constant hazard ratios. Solid black line indicates posterior median and filled bands credible intervals.

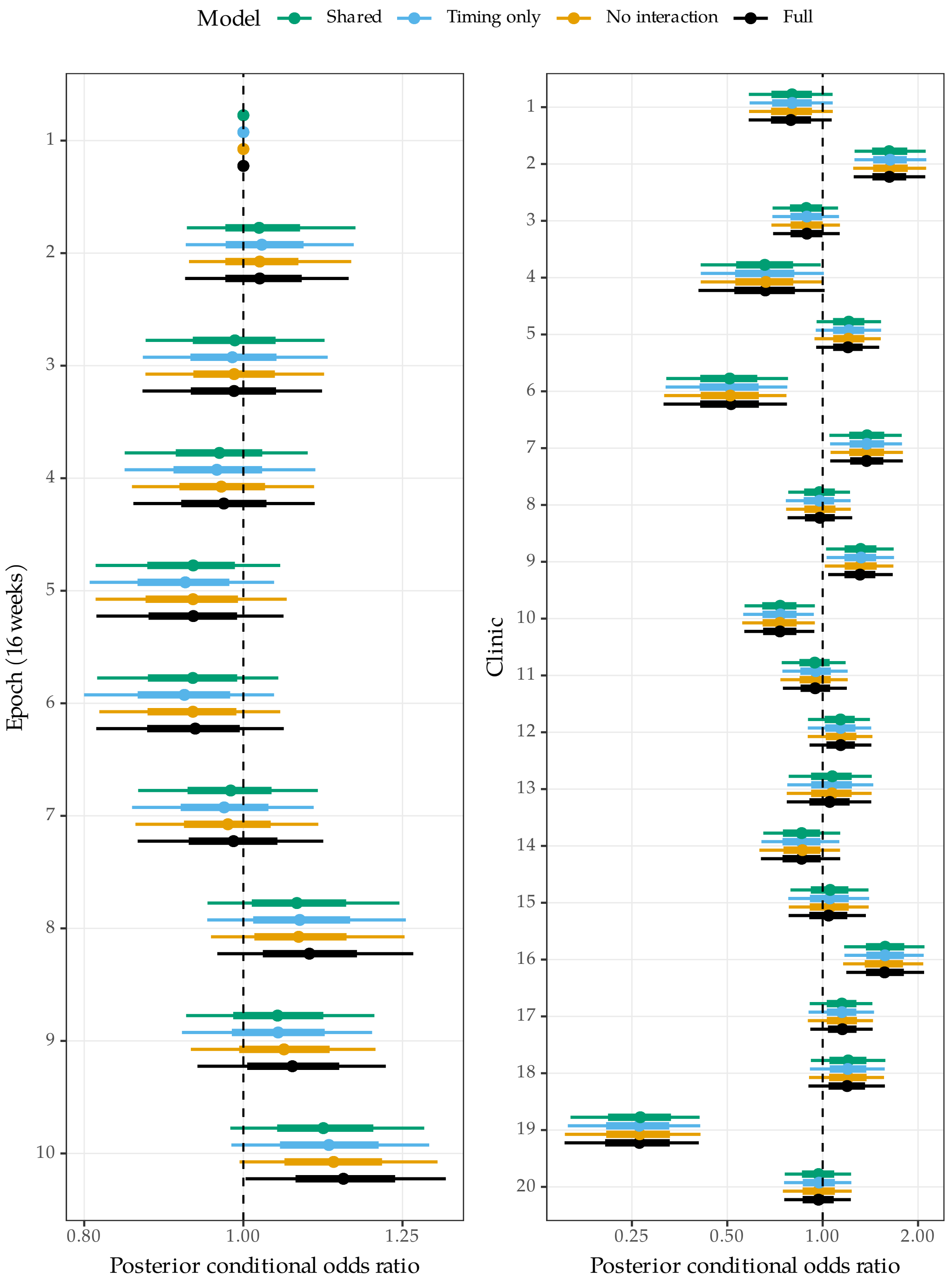

Figure S10: Estimated vaccination age, clinic, and epoch continuation ratios for day of vaccination amongst SMS eligible index vaccinations, by assumed model. Points – median, rectangles – 80% CrI, lines – 95% CrI.

5.1 Age-specific time-varying effect of message

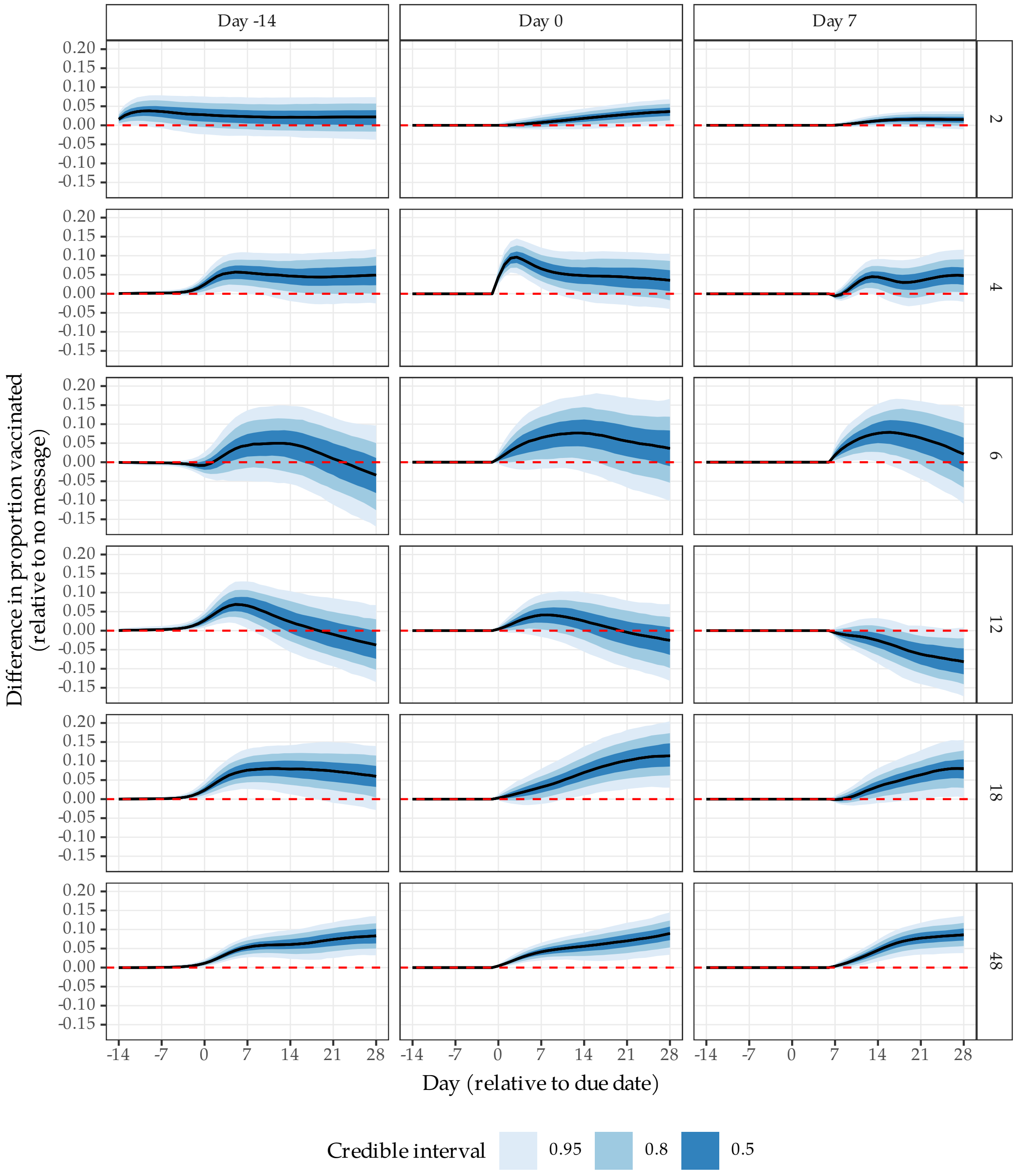

Figure S11: Posterior difference in cumulative incidence of vaccination by day relative to scheduled due date under a "timing-only" model with time-varying hazard ratios for a "typical" clinic (zero-effect). by vaccination age. Solid black line indicates posterior median and filled bands credible intervals.

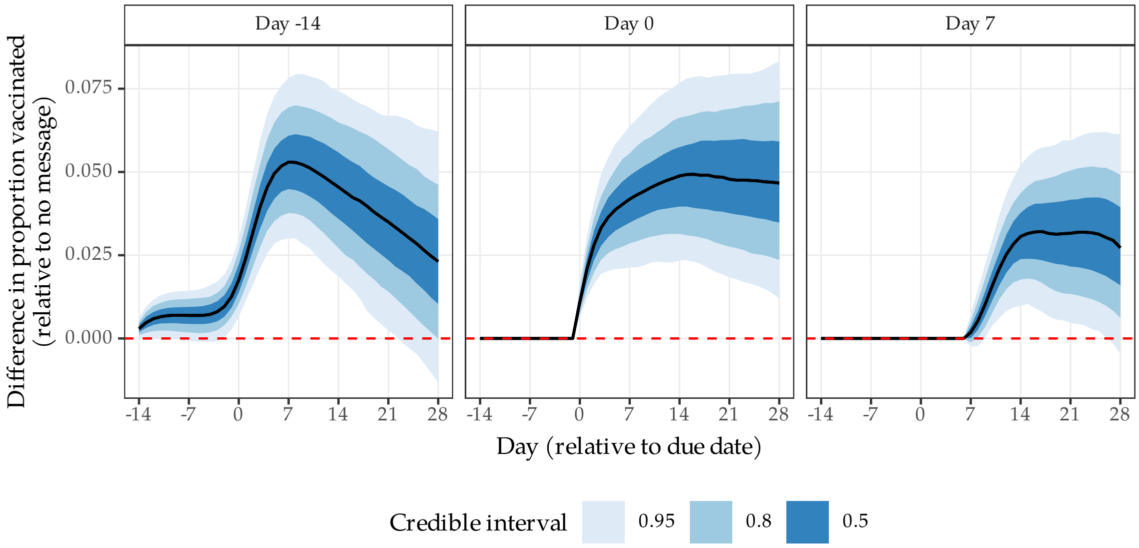

Figure S12: Posterior difference in cumulative incidence of vaccination by day relative to scheduled due date under a "timing-only" model with time-varying hazard ratios for a "typical" clinic (zero-effect) and simple average across all vaccination ages. Solid black line indicates posterior median and filled bands credible intervals.
